# Length-Dependent Transition from Extended to Folded Shapes in Short Oligomers of an Azetidine-Based α-Amino Acid: The Critical Role of NH···N H-Bonds

**DOI:** 10.3390/molecules28135048

**Published:** 2023-06-28

**Authors:** Dayi Liu, Jean-Xavier Bardaud, Zeynab Imani, Sylvie Robin, Eric Gloaguen, Valérie Brenner, David J. Aitken, Michel Mons

**Affiliations:** 1 Université Paris-Saclay, CNRS, ICMMO, 91400 Orsay, France; 2 Université Paris-Saclay, CEA, CNRS, LIDYL, 91191 Gif-sur-Yvette, France; 3 Université Paris Cité, Faculté de Pharmacie, 75006 Paris, France

**Keywords:** hydrogen bonds, conformational analysis, amino acids, peptides, azetidine, infrared spectroscopy, gas phase laser spectroscopy, quantum chemistry

## Abstract

Hydrogen bonds (H-bonds) are ubiquitous in peptides and proteins and are central to the stabilization of their structures. Inter-residue H-bonds between non-adjacent backbone amide NH and C=O motifs lead to the well-known secondary structures of helices, turns and sheets, but it is recognized that other H-bonding modes may be significant, including the weak intra-residue H-bond (called a C5 H-bond) that implicates the NH and C=O motifs of the same amino acid residue. Peptide model compounds that adopt stable C5 H-bonds are not readily available and the so-called 2.0_5_-helix, formed by successive C5 H-bonds, is an elusive secondary structure. Using a combination of theoretical chemistry and spectroscopic studies in both the gas phase and solution phase, we have demonstrated that derivatives of 3-amino-1-methylazetidine-3-carboxylic acid, Aatc(Me) can form sidechain–backbone N–H···N C6γ H-bonds that accompany—and thereby stabilize—C5 H-bonds. In the capped trimer of Aatc(Me), extended C5/C6γ motifs are sufficiently robust to challenge classical 3_10_-helix formation in solution and the fully-extended 2.0_5_-helix conformer has been characterized in the gas phase. Concurrent H-bonding support for successive C5 motifs is a new axiom for stabilizing the extended backbone secondary structure in short peptides.

## 1. Introduction

**Figure 1:**
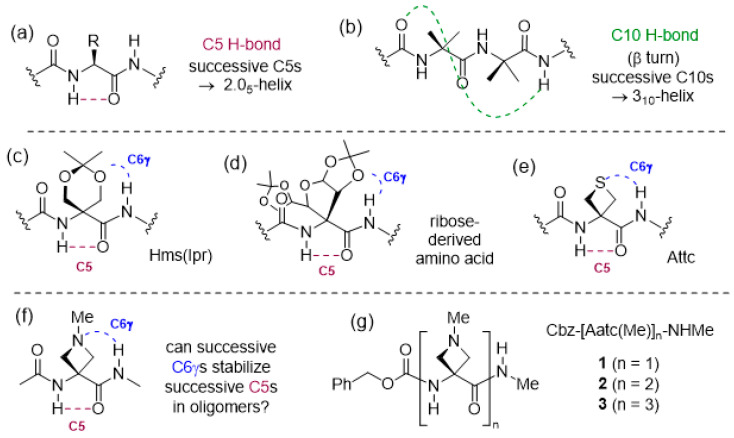
(**a**) The weak intraresidue C5 H-bond, forming the basis of the 2.0_5_-helix and (**b**) the more robust C10 H-bond, forming the basis of the 3_10_-helix. (**c**–**e**) Previously described examples of C6γ N–H···X interactions that accompany C5 H-bonds (X = O or S). (**f**) The combination of C5 and C6γ N–H···N interactions proposed in this work and (**g**) the compounds studied (**1**–**3**).

Nature uses hydrogen bonds (H-bonds) to organize a vast array of molecular architectures and to modulate biomolecular assemblies [1,2,3]. This is particularly evident in the area of peptides and proteins, where intramolecular H-bonds that are formed between backbone NH and CO motifs lead to the ubiquitous secondary structures of helices, turns and sheets [4,5,6]. In addition to these well-known interactions, other H-bonding modes are receiving increasing attention, including the intra-residue N–H···O=C backbone–backbone H-bond, often referred to as a C5 H-bond due to the size of the ring made by the non-covalent interaction [7] (Figure 1a). A number of studies have been carried out over the years in search for evidence for C5 structures in derivatives of single amino acids or small peptides; it transpires that the C5 H-bond is relatively weak and often cedes to alternative folded structures that benefit from stronger H-bonds [8,9,10,11,12,13,14,15,16,17]. Successive C5 interactions in a peptide sequence generate what is known as the 2.0_5_-helix, which (despite its name) constitutes a fully extended architecture. It was first noted as a plausible secondary structural feature for (Gly)_n_ sequences by Pauling [18], but remained largely overlooked for some time. Recently, arguments have been made for a stabilizing role for C5 interactions that accompany other non-covalent interactions in proteins [19], while a protein database analysis has revealed several dozen examples of extended segment stretching over three or four consecutive residues [20]. Considerable efforts have been made to identify the types of amino acids that may promote extended conformations in short homooligomers, proposed as molecular spacers [21], and the results have been comprehensively reviewed [20,22]. It has emerged that α,α-disubstituted amino acids in which at least one (but usually both) of the alkyl or aryl hydrocarbon side chains has two or more carbons are the most propitious for the adoption of 2.0_5_ helical conformations in homooligomers. It would appear that steric effects are largely at play, preventing the adoption of alternative folded conformations. It is of note that oligomers of less voluminous α,α-disubstituted amino acids, such as Aib [23,24] or its carbocyclic analogues Ac_n_c [25,26,27], have a strong preference to adopt 3_10_-helix structures (Figure 1b). While most work has been conducted in solution or solid states, a gas phase study of short Aib oligomers confirmed the intrinsic propensity of this amino acid to promote 3_10_-helical folding [28].

For oligomers of cyclic Aib derivatives, the influence of a side-chain oxygen atom specifically at the γ-position (using the α-amino acid nomenclature) on the conformational preferences has been observed on several occasions. Whereas short Ac_6_c oligomers were shown to adopt β-turn and 3_10_-helix conformations [29], the corresponding derivatives of *O,O*-isopropylidene-α-hydroxymethylserine, Hms(Ipr), preferred conformations in which intraresidue C5 H-bonds were accompanied by N–H···O (acetal) H-bonds, implicating a backbone amide NH(*i*) and a side-chain oxygen atom of residue (*i* − 1) (Figure 1c) [30]. This is referred to as a C6γ interaction, since the H-bond acceptor atom occupies the γ-position with respect to the α-amino acid core. In the crystal structure of a pentapeptide of a ribose-derived amino acid, a fully extended 2.0_5_-helix was evident with each C5 interaction accompanied by a C6γ N–H···O (acetal) H-bond, implicating the ring oxygen of each (*i* − 1) residue (Figure 1d) [31]. In contrast, for Ac_n_c oligomers with exocyclic ether functions whose oxygen atoms occupy the δ-position, the conformational preferences reverted to the helical mode; the pentapeptide of an Ac_3_c bearing two methoxymethyl substitutents adopted a bent 3_10_-helix in which one N–H···O=C interaction was replaced by a 10-membered ring N–H···O (ether) H-bond [32], while the trimer of Ac_6_c bearing a spiroacetal at the ring 3-position adopted a helical shape, devoid of backbone N–H···O=C interactions, stabilized only by intraresidue C6δ N–H···O(acetal) H-bonds [33]. 

Cyclic Aib derivatives incorporating a sulfur atom in the ring have also been studied. Short oligomers of Thp, the thioether analog of Ac_6_c with the sulfur in the δ-position, adopted regular 3_10_-helical conformations with no heteroatom implications [34], while a model compound study of Atc, a structural isomer with sulfur in the γ-position, suggested that intra-residue C5γ N–H···S interactions were the preferred side-chain involvement, leading to a locally folded geometry [35]. In contrast, using a combination of theoretical chemistry calculations, gas phase and solution phase studies, it was demonstrated that Attc, the four-membered thioether analogue of Ac_4_c, adopted a planar C5 conformation stabilized by inter-residue C6γ N–H···S interactions in the gas phase (Figure 1e). In the dimer, two successive C5-C6γ motifs led to a fully extended 2.0_5_-helix conformation. In the trimer, however, two local C5-C6γ motifs were separated by a turn feature, breaking the helical continuity [36].

Information is sparse regarding the behavior of cyclic Aib derivatives featuring an amine in the ring. Oligomers of 4-aminopiperidine-3-carboxylic acid, the aza analog of Ac_6_c with a secondary amine incorporated in the δ-position, adopted a helical conformation in acidic solution when the amines were protonated. On the other hand, in basic solution, the oligomers were devoid of H-bonds along the peptide backbone and it was suggested that each free amine might form a C7δ H-bond with the backbone amide NH (*i* + 1), although no role was indicated for an intraresidue C5 H-bond [37,38]. A heptamer of 3-amino-pyrrolidine-3-carboxylic acid (the natural product cucurbitin), with a secondary amine incorporated in the γ-position, was examined in solution using circular dichroism and was found to be more inclined to adopt a helical structure when the ring nitrogen was protonated, although an evaluation of other possible conformations was not disclosed [39]. 

In order to further assess the implications of concomitant side chain–backbone N–H···N H-bonding for the stabilization of a C5 conformation, we envisaged the study of derivatives of 3-amino-1-methylazetidine-3-carboxylic acid (Figure 1f). In a recent preliminary assessment of 4-membered ring heterocyclic α,α-disubstituted amino acid derivatives, we found that the C6γ N–H···N interaction is at least as strong as the C6γ N–H···S interaction shown by Attc [40]. While related azetidine-based α-amino acids have been incorporated into diverse molecular structures in drug discovery programs [41,42,43,44], information on their conformational behavior is almost non-existent. In a solitary study [45], tripeptides of structure Cbz-Ala-Aatc(R)-Ala-OMe (R = *t*-butyl or *t*-amyl) were shown to adopt principally β-turn conformations in solution. In the present work, we use a combination of theoretical chemistry and spectroscopic techniques in both the gas phase and solution phase to explore the conformational behavior of the short series Cbz-[Aatc(Me)]_n_-NHMe (n = 1–3) (compounds **1**–**3**) (Figure 1g), and compare it with the corresponding sulfur series Cbz-[Attc]_n_-NHMe, (n = 1–3).

## 2. Results

### 2.1. Gas Phase Conformational Analysis

#### 2.1.1. Theoretical Landscapes

The gas phase conformational landscape of compound **1** was previously analyzed [40]. It was found to consist of the following three backbone families: the C5-C6γ, C7 and δ conformational families. Respectively, these structures are based on the following: (i) an extended backbone (labelled 5 for short) stabilized by an intra-residue C5 and an inter-residue C6γ H-bond, implicating N1H and N2H, respectively; (ii) a folded backbone built around a C7 H-bond (7 for short) formed by N2H; and (iii) a semi-folded structure (δ for short), wherein the amide N2H is oriented towards the preceding nitrogen in a so-called π-amide (π_am_) bond. In the present work, the assessment of the conformational landscape of **1** was extended to include the solution phase, where the same conformer families were found. 

**Figure 2:**
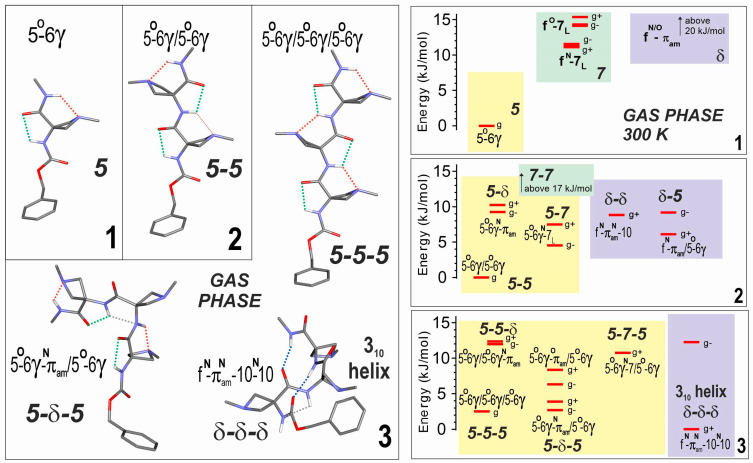
(**left**) Gas phase geometry-optimized structures obtained by quantum chemistry (RI-B97-D3(BJ)-abc/def2-TZVPPD level of theory) of the most stable backbone conformations of compounds **1**–**3**. Bold labels indicate backbone types. The conformational nomenclature indicates the H-bonding status of each NH along the backbone (5, 6γ, 7, 10, as a short notation for C5, C6γ, C7, C10 H-bonds; f for free; π_am_ for a π–amide interaction with the preceding amide nitrogen). The ring puckering of each Aatc(Me) residue is indicated as a superscript (N or O) depending on whether the azetidine N atom is leaning towards the NH or CO group of its residue, respectively. Each structure is given with a *gauche+* conformation of the Cbz cap. The interactions of NH groups are identified by colored dotted lines: green C5, red C6γ, blue C10, grey π_am_. (**right**) Gas phase theoretical conformational landscape of compounds **1**–**3** at 300 K. Conformations in the same colored region share the same type of local backbone on their first (N-terminal side) residue. For the sake of simplicity, only *gauche* conformations were considered for building up the landscape. Each level shown corresponds to an enantiomeric pair: for the extended (achiral) backbones (e.g., 5-5-5), it is composed of both *gauche*+ and *gauche*− Cbz orientations and globally noted *g*; for the other (chiral) backbones (i.e., having at least one 7 or δ local backbone, such as 5-7-5 or δ-δ-δ), the enantiomeric pair is composed of one enantiomeric backbone conformation (e.g., 5-7_L_-5, 5-7_D_-5, δ-δ-δ, or δ′-δ′-δ′), combined with a given (*gauche*+ or *gauche*−) Cbz orientation, together with its mirror image conformation. For the sake of clarity, in the landscape picture, a single enantiomeric form is shown, namely that whose backbone enantiomer form comprises a 7_L_ or a δ local backbone conformation combined with the corresponding Cbz orientation.

The analyses of the conformational landscapes of compounds **2** and **3** were carried out in the gas phase at 300 K since previous studies had shown that this temperature provides a suitable agreement with the population distributions measured in a supersonic expansion [36,40,46,47]. The results were compared with those already known for **1** (Figure 2) and collectively, they indicated the significant evolution of the conformational preferences with the number of residues in the oligomer. 

The dominant conformer (5-5) of compound **2** displayed two successive C5-C6γ local backbone preferences (Figure 2). The next most stable conformations were more energetic by more than 4 kJ·mol^–1^ and implicated the replacement of the 5 motifs by a 7 or a δ backbone at the second residue (5-7 or 5-δ), at the first residue (δ-5), or at both residues (δ-δ). This latter structure formed a β-turn, characterized by a C10 H-bond implicating N3H; despite the additional stabilization provided by the H-bonding, this folded backbone remained considerably more energetic than the extended form, presumably due to the backbone distortions required to enable the H-bond formation.

Competition was found to be more open in compound **3**, since the fully extended form (5-5-5) was found to be challenged by two other low energy forms (Figure 2). The first was a half-extended form (5-δ-5), in which the central 5 motif of the fully extended form was replaced by a δ conformation at the expense of the breakage of one C6γ bond. The second featured three successive δ motifs (δ-δ-δ) and exhibited two parallel C10 H-bonds implicating H3H and N4H, corresponding to an incipient 3_10_-helix. In this case, however, it should be noted that a *gauche+* orientation of the Cbz cap was evident, enhancing stability through ancillary interactions involving the phenyl ring and the side chains of the second and third residues (see Supplementary Materials details Section S1.2). 

#### 2.1.2. Gas Phase Laser Spectroscopy and Quantum Chemistry Calculations

**Figure 3:**
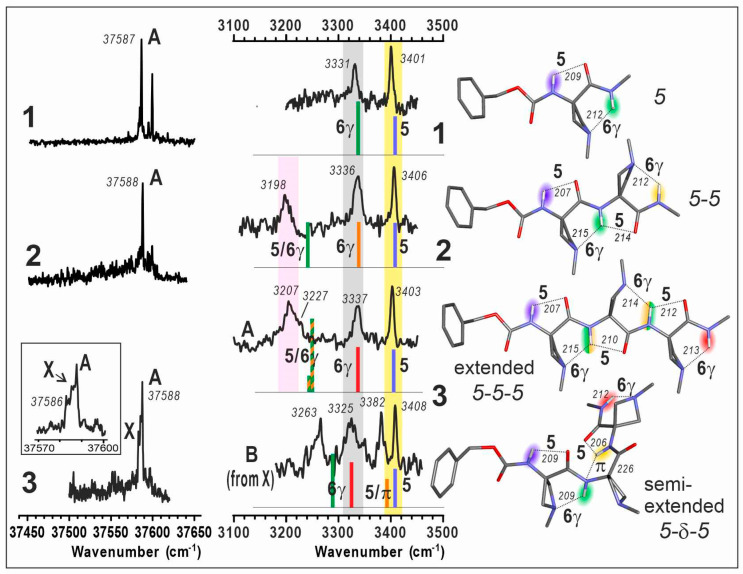
(**left**) Gas phase mass-selected UV spectra of jet-cooled compounds **1**–**3**. An enlargement of the features for **3** is given in the inset. (**middle**) Gas phase IR spectra (black), recorded on the UV band A for each compound **1**–**3** together with that obtained for a secondary conformer B of compound **3** (obtained as a minor contribution in the spectrum recorded on the UV band X; see SI, Section S2 for procedure details). For comparison, theoretical IR spectra (sticks; DFT-D method at the RI-B97D3(BJ/abc)/def2-TZVPPD level of theory) of the Cbz-cap *gauche*+ rotamer of the lowest energy conformations are presented; the stick color code (rainbow colors from blue to red) indicates the position of the NH bond from the N- to C-terminal. The pastel bands (yellow, grey and pink) indicate the experimental spectral features of the C5, C6γ bonds and C5/C6γ motifs, respectively. (**right**) Corresponding calculated lowest energy extended conformations of **1**, **2**, **3** and the second most stable semi-extended conformer of **3**. H-bonding distances are given in pm. UV and IR spectra of **1** are adapted from Ref. [40].

The R2PI UV spectra in the first near UV absorption region of the benzyl group were composed of narrow lines, from which the conformational populations could be distinguished (Figure 3, left). IR absorption spectra in the NH stretch region of each UV-distinct conformer were then recorded by IR/UV laser spectroscopy (Figure 3, middle). This method, coupled to laser desorption for vaporization and supersonic expansion for efficient cooling, enabled selective access to the IR spectrum of each conformer observable in the expansion, allowing a direct comparison with calibrated quantum chemistry calculations, and therefore an often unambiguous assignment of the conformer population.

The UV spectra of compounds **1**–**3** all showed an intense UV band (labelled A) at about 37,588 cm^–1^, suggesting that in these species, the Cbz caps experience a very similar local environment, and in particular similar N terminus peptide conformations. 

In compound **1**, a satellite band located 22 cm^–1^ higher in energy was previously assigned to the same conformer as band A, as suggested by the same IR/UV spectra obtained on these two bands. In addition to band A, compound **2** exhibited a similar UV satellite band for which the same assignment was made, namely a vibronic band of the same conformer as band A. Compound **3** exhibited a broader band A, accompanied by a red-side shoulder (labelled X), which anticipated a more complex landscape. 

The IR spectra recorded on band A of compounds **2** and **3** shared two features, i.e., two isolated bands at ca. 3336 and 3406 cm^–1^. These bands, already observed in the spectrum of **1** (Figure 3, middle, grey and yellow regions), were assigned to the C6γ and C5 interactions present in the 5 conformer of this species [40]; this assignment was further supported by the sustained presence of this C5-C6γ motif across a series of similar single-residue heterocyclic amino acid derivatives, when varying the heteroatom from N to S to O [40]. In compounds **2** and **3**, however, a new, broad band appeared closer to the red region at ca. 3200 cm^–1^ (Figure 3, middle, pink region), suggesting the presence of strong H-bonding, in addition to isolated C6γ and C5 interactions. Comparison with quantum chemistry calculations of extended forms (5-5 and 5-5-5) provided a fair agreement of these red-shifted bands to the presence of one and two bifurcated H-bonds, in **2** and **3** respectively; in these conformers, an NH bond is involved simultaneously in a C5 and C6γ H-bond, leading to a significant red shift, reproduced (albeit slightly underestimated) by calculations. The “missing” IR band of **3** (which should show four NH stretch bands) can be assigned to the vibrational coupling of the N2H and N3H moieties into a symmetric and an antisymmetric component, with the former being much less intense and probably responsible for the shoulder observed at 3227 cm^–1^. These gas phase results demonstrate the persistence of the C5-C6γ motif as a prevalent local planar backbone conformation in short oligomers of Aatc(Me), for which two or three consecutive motifs lead to the globally extended architecture of a 2.0_5_-helix.

Interestingly, the IR spectrum recorded from the UV band X of **3** was found to be of hybrid nature (see Supplementary Materials, Section S2) exhibiting two components, one of them being that of conformer A (as recorded on the UV band A) and the second one was reconstructed by spectral subtraction and assigned to a minor conformer labelled B. This latter spectrum comprised the two bands mentioned above for **1**, corresponding to isolated C5 and C6γ bands, but the two remaining bands were found to differ significantly from those of the extended form A, suggesting a different conformation for the central part of the molecule. Comparison with the semi-extended 5-δ-5 form, where the second residue adopts a δ conformation, provided a good agreement; the rupture of the C5 interaction of the 5-6γ motifs in A leaves a C6γ bond and forms a bifurcated π_am_/C5 bond instead, for N2H and N3H moieties, respectively. This demonstrated the presence of a second conformer in **3**, an observation further supported by the very similar stabilities for the extended 5-5-5 and semi-extended 5-δ-5 forms (Figure 2, right). 

Regarding compound **3**, the presence of red-shifted IR bands below 3350 cm^−1^ in the spectra of conformers A and B (Figure 3, pink and grey regions) cannot be accounted for by a 3_10_-helical (δ-δ-δ) conformation. Indeed, this form exhibits only two relatively weak H-bonding C10 interactions (Figure 2), whose signatures are expected around 3400 cm^−1^ [46]. The absence of this conformation in the present gas phase experiment, despite favorable energetics, will be discussed further in Section 3. 

### 2.2. Conformational Analysis in Chloroform Solution

#### 2.2.1. Theoretical Landscapes

**Figure 4:**
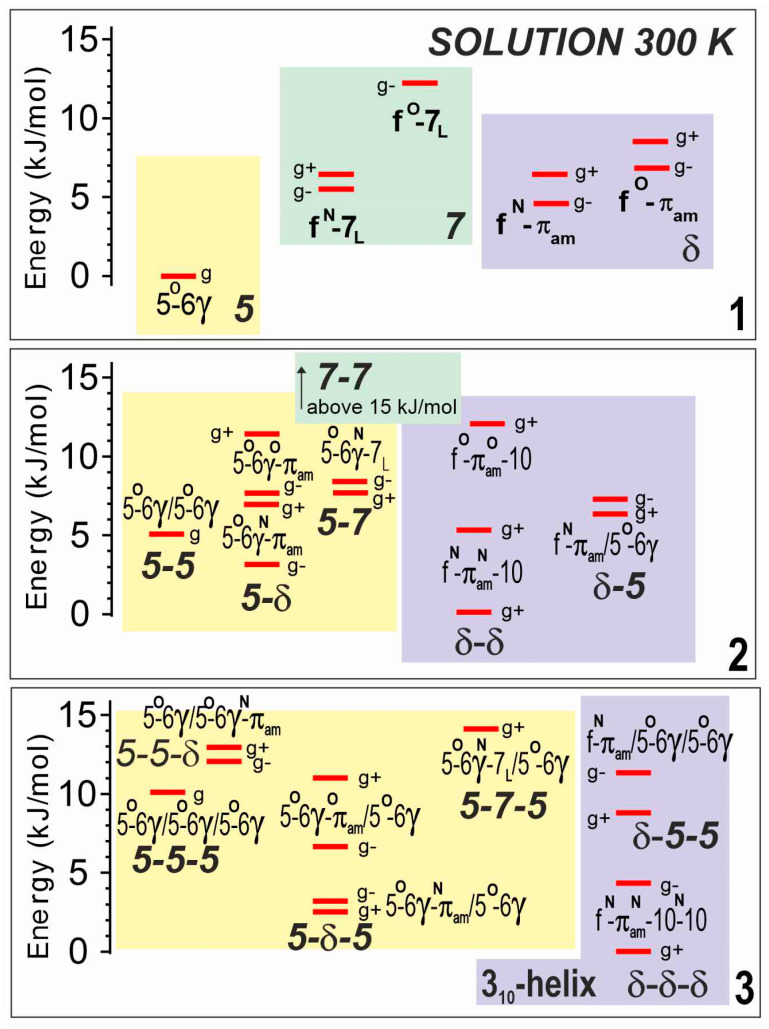
Theoretical landscapes of compounds **1**–**3** in a chloroform solution at 300 K, as obtained at the DFT-D level of theory with a solution modeled by a polarizable continuum (RI-B97D3(BJ-abc)/TZVPPD + COSMO model). Same caption details as for Figure 2. Lowest energy structures are given in Section S3.1 of the SI.

The theoretical analyses of compounds **1**–**3** were carried out in solution, by reoptimizing all the gas phase structures in a solvent environment, modeled by a polarizable continuum. Not surprisingly, the landscapes (Figure 4) displayed the same backbone families as in the gas phase, with very similar structures, including the three basic backbones (5, 7 and δ) for **1**, and combinations thereof for **2** and **3**, leading to fully extended (5-5 and 5-5-5), semi-extended (5-δ and 5-δ-5) and turn or helical structures (δ-δ and δ-δ-δ). The relative stabilities of these families, however, were found to vary significantly. The fully extended form appeared to be less and less favored as the number of residues increased (i.e., following the series **1**→**2**→**3**), whereas the two other families were predicted to be more populated, with a slight preference for the turn/helical structures, in agreement with the larger dipole moment of the latter.

#### 2.2.2. Solution-State IR Spectroscopy and Quantum Chemistry Calculations

**Figure 5:**
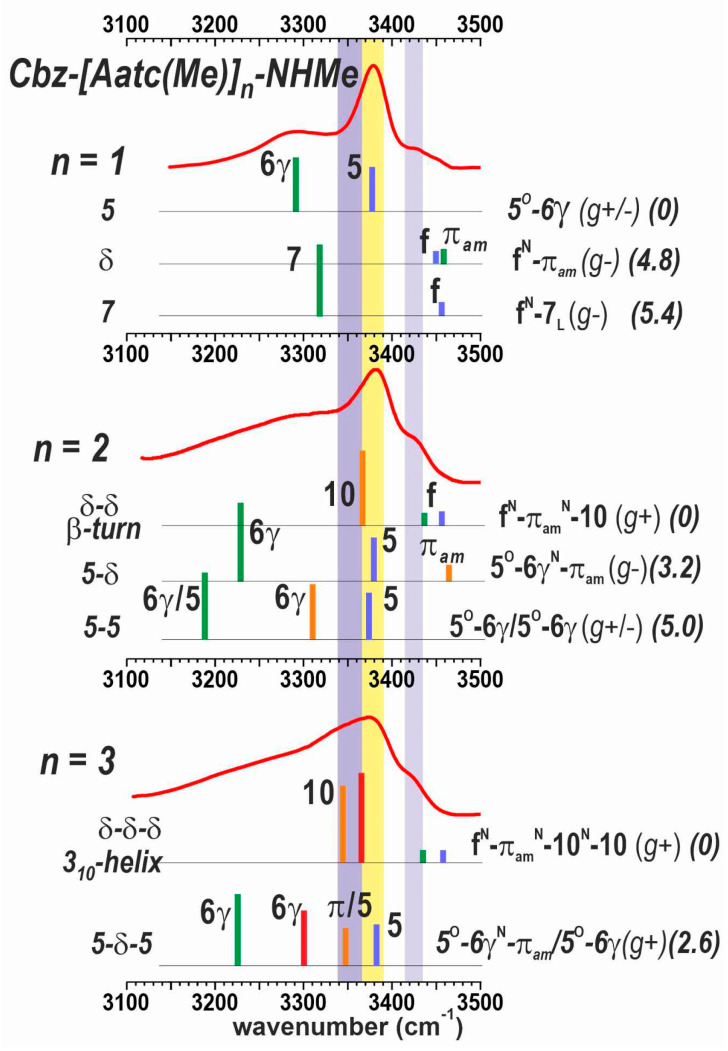
Solution-state IR spectra (red trace) of compounds **1**–**3** (5 mM in chloroform) and calculated IR spectra (colored vertical bars) of their most energetically relevant conformations (300 K energetics in kJ·mol^–1^ between parentheses). The NH stretches are localized in each residue and the residue position from N to C terminus is rainbow color-coded from blue to red. The colored spectral regions indicate the typical locations of diagnostic spectral features: nearly free (π_am_) NH in a δ conformation (lilac), NH engaged in a C5 H-bond (yellow) or in a C10 H-bond (mauve). The spectrum of **1** is adapted from Ref. [40]. A more complete set of theoretical spectra is provided in Section S3.2 of the SI.

The IR absorption spectra of compounds **1**–**3** were recorded in chloroform. No concentration-related effects were observed in the range 0.5–10 mM (see SI, Section S5.1), indicating the intramolecular nature of any non-covalent interactions. The absorbances in the amide NH stretch region are shown in Figure 5 for 5 mM solutions. Within the series, notable differences were observed. Two main bands were observed at 3380 and 3290 cm^−1^ for **1** and were assigned to C5 (yellow region) H-bonded N1H and C6γ H-bonded N2H, respectively, in the planar structure corresponding to the principal gas phase 5 conformation. Two weak high-frequency bands were also evident in the 3430–3450 cm^−1^ region, and were assigned to minor contributions from δ and 7 conformations, whose free NH bonds are expected to be found in this region [35,40].

The IR absorption spectrum of **2** exhibited the following three distinctive features: (i) the intense band at 3380 cm^–1^ (yellow region) was present but dissymmetric, suggesting a shoulder on its red side (mauve region); (ii) a higher frequency band at 3430 cm^–1^ (lilac region) accompanied by a blue-side buttress up to 3460 cm^–1^; and (iii) a broad feature in the red region centered at 3280 cm^–1^ and extending down to 3150 cm^–1^. The blue band was informative of the presence of conformations having free or nearly free NHs. The quantum chemistry calculations in the previous section (Section 2.2.1) suggested that three conformer families with different types of backbone are found in a 5 kJ·mol^–1^ range and may thus be expected to be populated in solution, including the extended form (5-5), the semi-extended form (5-δ) and the β-turn (δ-δ) respectively. The two latter forms present a nearly free N3H and a free N1H, respectively, which would account for the presence of the 3430 cm^–1^ absorption. The C10 H-bond N3H of the β-turn also accounted for the red shoulder of the 3380 cm^–1^ band (mauve region). Overall, the C5 band at 3380 cm^–1^ (yellow) indicated that the first residue of **2** exhibited a C5 conformation in some of its populated forms, but the other features (mauve and lilac), together with the absence of significant absorption in the 3200 cm^–1^ region (where the bifurcated C5-C6γ H-bonded N2H absorption is expected), suggested that the extended form was challenged by alternative conformations.

The IR spectrum of **3** (Figure 5) continued the trend set by compound **2**. A significant absorption stretched across the 3340–3380 cm^−1^ range (yellow and mauve regions) and was accompanied by a blue band at 3430 cm^−1^ (lilac region). A long and near-featureless red-side tail-off extended down to 3150 cm^−1^. Comparison with the calculated IR spectra of the most stable forms found by quantum chemistry (Section 2.2.1) showed that the experimental absorption features are satisfactorily explained by the presence of the following two main conformations: (i) the semi-extended form (5-δ-5) that had made a minor contribution in the gas phase, and (ii) the incipient 3_10_-helix conformation (δ-δ-δ), implicating three successive π_am_ interactions and two successive C10 H-bonds.

#### 2.2.3. Solution-State ^1^H NMR Spectroscopy

**Figure 6:**
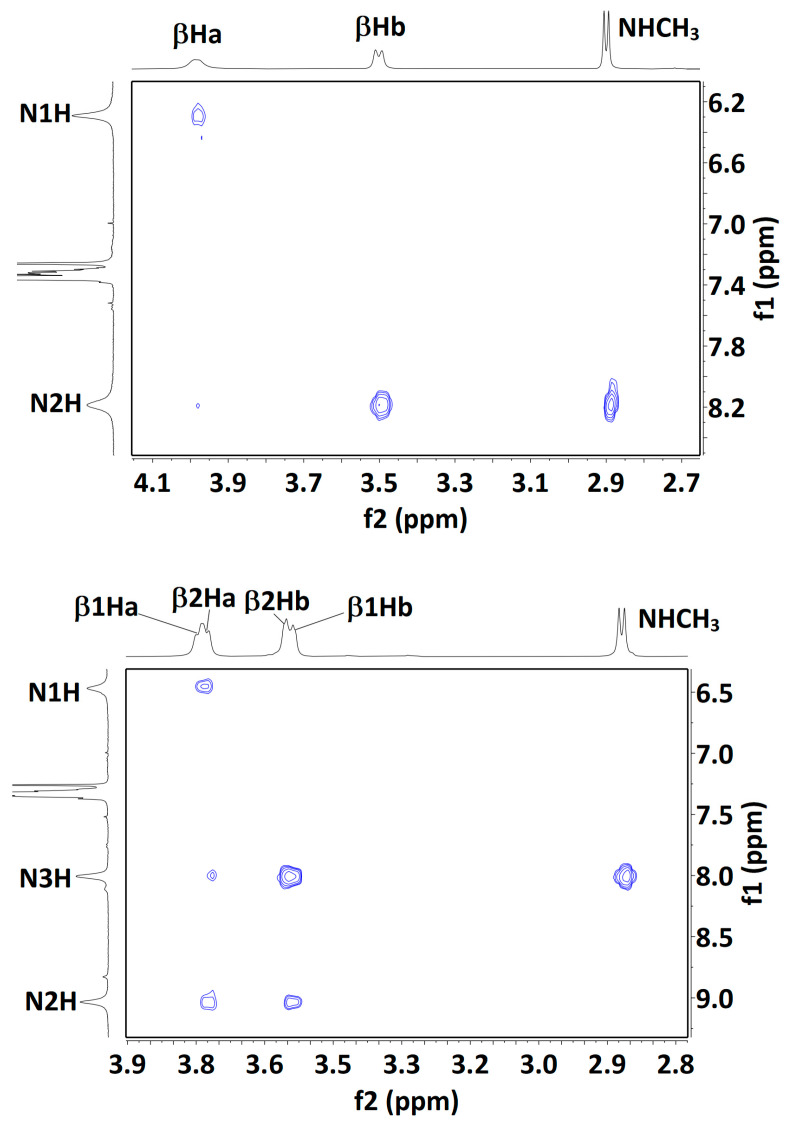

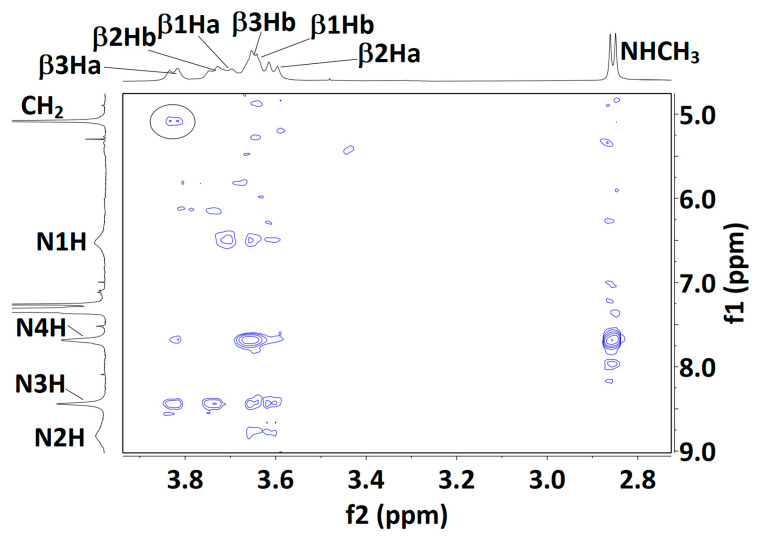
Solution-state (20 mM in CDCl_3_) ^1^H NOESY correlation maps for compounds **1** (**top**), **2** (**middle**) and **3** (**bottom**). Assignments for the βH signals of compound **3** are plausible but not unambiguous, with the exception of the lowest field signal, assigned to β3Ha; its correlation with the Cbz benzyl signal is indicated (black oval). For each Aatc(Me) residue, the βHa and βHb hydrogens are located on the NH and the CO side of the azetidine ring, respectively.

The presence of more than one solution-state conformation makes the interpretation of NMR data difficult, but for completeness, we carried out some experiments using solutions in CDCl_3_ (see SI, Sections S5.1 and S5.2). Compound **1** showed low-field signals for the carbamate N1H and amide N2H protons (δ = 6.35 and 8.24 ppm, respectively) with low-to-moderate DMSO-*d*_6_ titration coefficients (∆δ = +0.29 and −0.42 ppm for 10% added DMSO, respectively). These 1D data were entirely compatible with the predominance of the 5 conformer, but did not rule out minor contributions from δ and/or 7 conformers. A NOESY experiment (Figure 6) showed strong correlations between N2H and both the methylamide and the βHb protons, while N1H showed a medium correlation with the βHa protons. Here again, these data can be explained in terms of a major 5 conformer but do not exclude contributions from the δ or 7 conformer families.

In compound **2,** the signals for carbamate N1H and amide N3H (δ = 6.14 and 7.94 ppm, respectively) were both slightly more shielded than the corresponding terminal NH proton signals in **1**. Of note, the signal for the internal N2H of **2** was strongly deshielded (δ = 9.07 ppm), consistent with the bifurcated three-center H-bonded system of the extended 5-5 conformation. DMSO-*d*_6_ titration coefficients were low-to-moderate for all three NHs (∆δ = +0.58, −0.62 and −0.33 ppm, for N1H, N2H and N3H, respectively), suggesting considerable on-average H-bonded characters for all three protons. The lower effect for N3H may be indicative of a contribution from the β-turn conformer, in which this amide is H-bonded while the other two are not. A NOESY experiment (Figure 6) showed a correlation map with only local interactions, similar to those observed for **1**. Specifically, N1H showed a medium correlation with β1Ha protons; N2H showed medium correlations with β1Hb and with the β2Ha protons; N3H showed stronger correlations with β2Hb and with the methylamide protons. Interestingly, the weak correlation between N3H and the β2Ha proton signals may be indicative of a contribution from a 5-δ conformer, in which the N3H–β2Ha distance is similar to the N3H–β2Hb distance (d_H-H_ = 3.71 and 3.79 Å, respectively). Collectively, these NMR data are compatible with the presence of the fully extended form (5-5) accompanied by the semi-extended (5-δ) and β-turn (δ-δ) conformations. 

NMR data for compound **3** were more difficult to interpret. The carbamate N1H and amide N4H signals remained deshielded (δ = 6.38 and 7.70 ppm, respectively), while N2H and N3H were more strongly deshielded (δ = 8.80 and 8.45 ppm, respectively). N1H and N2H appeared as very broad peaks, indicative of an exchange process or interconversion between several conformations. DMSO-*d*_6_ titration coefficients remained moderate for the three amide NHs (∆δ = −0.51, −0.58 and −0.60 ppm, for N2H, N3H and N4H, respectively) and perceptibly higher for carbamate N1H (∆δ = +0.95 ppm). This latter observation may be the result of a weakened C5 H-bond and/or contributions from conformers in which this NH is free, including the 3_10_-helix (δ-δ-δ) conformer. The NOESY experiment (Figure 6) produced only a poorly defined correlation map, suggesting considerable conformational interconversion. Furthermore, meaningful interpretation was thwarted by extensive overlapping of the βH signals, precluding unambiguous assignments; only the β3Ha signal was identified with confidence. It showed a medium correlation with N3H and a weaker correlation with N4H, compatible with a local C5/C6γ conformation at residue 3. However, the β3Ha signal also showed a weak correlation with the Cbz benzyl proton signal, which can only be explained by the presence of a folded 3_10_-helix conformer (d_H-H_ = 2.91 Å).

## 3. Discussion

The gas phase experiments provided convincing evidence for stable extended conformations in all three capped Aatc(Me) derivatives **1**–**3**. The successive locally planar C5-C6γ motifs override the options to fold into more compact structures, resulting in a dominant extended backbone conformation of the 2.0_5_-helix. These architectures are globally stabilized through the succession of the cooperative intra-residue C5 backbone and vicinal N–H···N C6γ H-bonds. 

The exclusive detection of extended forms in **1** and **2** is indeed consistent with the theoretical stabilities suggested by the conformational landscape analysis (Figure 2). This analysis also suggested that the helical form of high energy in **2** is more stabilized in **3** to the point where it should be observable. For compound **3**, however, the anticipated 5-5-5 and 5-δ-5 conformers were detected, whereas the δ-δ-δ helical form was not. The absence of a helical signature can be ascribed to several causes. A basic explanation could be given by the limited precision of the theoretical energetics, which is typically of the order of a few kJ·mol^−1^. Other explanations are of an experimental nature. One can for instance invoke a kinetic trapping effect (see SI, Section S1.3) that takes place in the expansion. The helical form is quite compact (see SI, Section S1.2) and is therefore entropically disfavored compared to its more floppy competitors at the high temperatures encountered following laser desorption. The rapid drop in the interconversion rate between conformers in the supersonic expansion, due to the presence of high interconversion barriers between very different backbones (helical vs. extended), would lead to the freezing of the conformational populations corresponding to high temperatures, and thus a weak population of helical structures. A second possible experimental explanation may lie in the UV spectroscopy measurements. As noted above, the helical form owes its specific stability to ancillary interactions involving the Cbz phenyl ring with either a CβH_2_ methylene group of the 2nd residue (CH···π interactions) or the N atom lone pair of the 3rd residue (CH···N interactions) (see SI, Section S1.2 for details). The phenyl ring UV spectrum might be strongly perturbed, with a more shifted UV transition than usually expected, and hence located in a region not yet investigated. Alternatively, the existence of a short lifetime for the S_1_ excited state might hamper the photoionization step of the R2PI process. Such dynamical processes in S_1_ have been documented, for instance in toluene–water clusters [48] or phenol–ammonia clusters [49], and ascribed in this latter case to a non-adiabatic transition to a nearby πσ* state in OH···NH_3_ geometry. Finally, reactivity in the gas phase ion causing fragmentation, in a similar way as was observed as a partial process for compound **2** and for the detected conformers of **3** (see Section 4.2), may occur more drastically in this conformer, due to a favorable interaction between the charged phenyl and the N atom lone pair.

The solution phase experiments showed a more evolutive conformational behavior along the series **1**→**2**→**3**, in agreement with the suggestions emanating from the theoretical landscapes (Figure 4). The predominant conformation of **1** is the planar form (5), stabilized by the robust constituent C5-C6γ motif. In compound **2**, both IR and NMR data pointed to the presence of the extended form (5-5), the semi-extended form (5-δ) and the β-turn (δ-δ). In compound **3,** the fully extended form (5-5-5) is not competitive and gives way to two main conformers, the semi-extended form (5-δ-5) and the 3_10_-helix (δ-δ-δ). It emerges from these studies that local C5-C6γ motifs are present on the conformational landscape of all three compounds—conformers 5 (for **1**), 5-5 and 5-δ (for **2**); 5-δ-5 (for **3**)—but that successive motifs, forming a 2.0_5_-helix implicating bifurcated C6γ/C5 H-bonds, are less competitive than alternative partially folded structures.

The above observations suggested that a reexamination of the solution-state IR spectra of the series of sulfur derivatives Cbz-[Attc]_n_-NHMe, (n = 1–3) [36] might be pertinent, notably in the light of quantum chemistry calculations performed on these molecules in a solvent environment modelled by a polarizable continuum (See SI, Section S4). These calculations suggest that, in solution, the helical forms are considerably more energetically favorable than in the gas phase and should, therefore, compete with extended forms.

**Figure 7:**
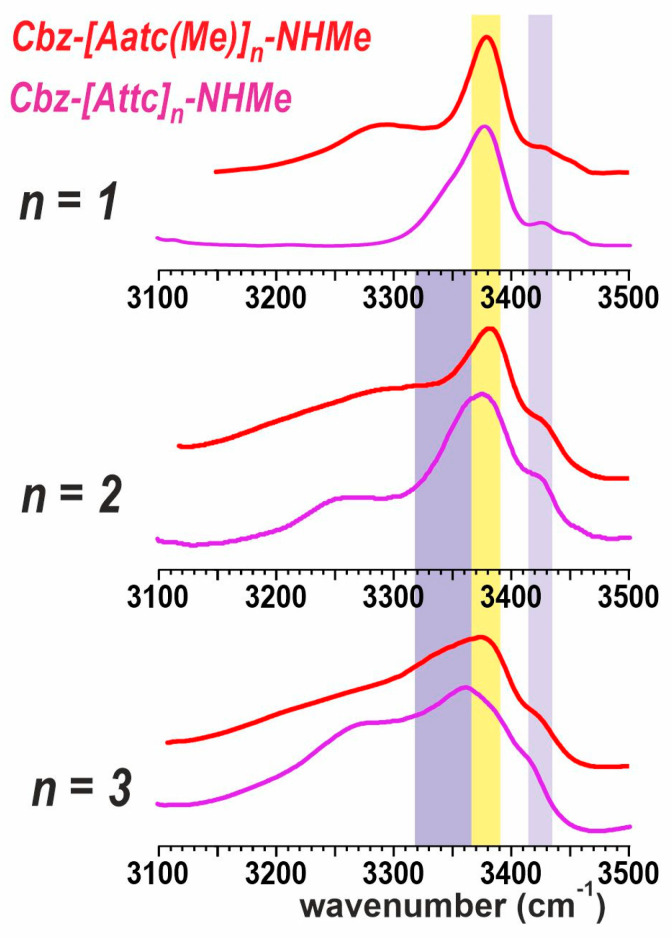
Solution-state IR spectra of compounds **1**–**3** (red traces) compared to their Attc counterparts (magenta traces). The colored spectral regions indicate the typical locations of diagnostic spectral features: nearly free (π_am_) NH in a δ conformation (lilac region), NH engaged in a C5 H-bond (yellow region) or in a C10 H-bond (mauve region). The spectrum of **1** is adapted from Ref. [40] and those of Attc compounds from Ref. [36].

The experimental solution-state IR spectra of the Cbz-[Attc]_n_-NHMe series and the Cbz-[Aatc(Me)]_n_-NHMe series (**1**–**3**) are compared in Figure 7. Monomer derivatives (n = 1) both show the predominance of a C5-C6γ motif, for which the C5 NH absorption is at ca. 3380 cm^−1^, while the C6γ NH absorption varies with the heteroatom involved (3290 cm^−1^ for Aatc(Me); 3350 cm^−1^ for Attc). Small bands in the range 3430–3450 cm^−1^ are indicative of minor contributions from other higher-energy conformers [40]. For the dimer derivatives (n = 2), the now-plausible C10 H-bonded (δ-δ) conformer would give rise to an NH absorption at ca. 3350 cm^−1^ (Figure 7, mauve region). This is more evident for the Attc derivative than for the Aatc(Me) derivative **2**, in agreement with the theoretical calculations that suggest the δ-δ conformer is energetically competitive with the 5-5 conformer for Cbz-[Attc]_2_-NHMe. For the trimer derivatives, the C5 H-bonding (yellow region) of the semi-extended form (5-δ-5) still persists for the Aatc(Me) derivative **3**, but has diminished to a shoulder in the case of Cbz-[Attc]_3_-NHMe, for which the C10 signature (mauve region) of the 3_10_-helix is more evident, again in agreement with the theoretical calculations.

This comparison suggests that, in solution, the transition from planar C5-C6γ motifs to folded, helix-enabling C10 motifs occurs more readily with Attc derivatives than with Aatc(Me) derivatives. It is worth recalling here, to keep these observations in perspective, that short oligomers of the carbocyclic analogue, Ac4c, show no evidence at all for adopting conformers that include C5 H-bonds [26,27]. This highlights the primordial role of the C6γ H-bond formed by the heteroatoms of Aatc(Me) and of Attc for the stabilization of C5 conformers.

In conclusion, this work provides the first detailed examination of the conformational preferences of short oligomers of an azetidine-derived α-amino acid. The demonstration of the fully extended 5-5-5 conformer of compound **3** in the gas phase is the first example of a 2.0_5_-helix whose stability relies on cooperative hydrogen bonding rather than steric effects. In solution, the fully extended 2.0_5_-helix gives way to folded conformations, one of which is the classical 3_10_-helix, but the other of which is a semi-extended conformer in which two separate C5-C6γ motifs prevail. This suggests that a local C5-C6γ motif may be sufficiently robust to serve as a localized planar “spacer” feature in larger peptides with more diverse primary sequences, and as such contributes a useful tool for the design of peptide architecture.

## 4. Materials and Methods

### 4.1. Theoretical Chemistry

Capitalizing on the analysis of **1**, the conformational landscapes of compounds **2** and **3** were reconstructed in the present work both in the gas phase and in solution. The conformational analysis was carried out by building up a juxtaposition of two and three local conformational preferences of compound **1** (one of each of the three conformations discussed above). These structures were then optimized at a quantum chemistry level (DFT-D), previously validated on compound **1**, for which the theoretical predictions (energetics and scaled vibrational spectra) provided a satisfactory counterpart to experiment in both the gas phase and solution. For the sake of simplicity, only *gauche* conformations of the Cbz cap were considered, since their *trans* counterparts are usually slightly less stable and their orientation does not favor stabilizing interactions with side-chain motifs. Likewise, only *trans* conformations of the carbamate moiety were considered. 

The structures were first optimized in the gas phase, at the B97-D3 level of theory [50] using Becke–Johnson damping and the three-body term options (RI-B97-D3(BJ)-abc) with a def2-TZVPPD basis set [51,52], using the *jobex* module of the Turbomole package [53]. The resolution-of-identity (RI) approximation [54] and the associated auxiliary basis [55,56] were also used. The normal modes, numerical harmonic frequencies and IR absorption strengths were calculated at the same level of theory using the *numforce* module. Gibbs energies were obtained at both 0 and 300 K, using the *freeh* module of Turbomole. A series of benchmark studies [57,58] have shown that this level of theory provided a satisfactory agreement between gas phase populations and theoretical predictions. 

Structures, energetics and frequencies in solution were carried out using the Conductor-like Screening Model approximation (COSMO) [59], available in the Turbomole package, where the solvent is modelled by a dielectric continuum of permittivity ε (4.81 for chloroform). 

For comparison with experimental spectra in the NH stretch region, the harmonic frequencies were scaled by factors previously determined to provide a fair agreement with the experiment (typical precision of ca. 20 cm^−1^) on compound **1**, namely 0.9780 and 0.9685, for the gas phase and the solution, respectively [35,36].

### 4.2. Gas Phase Experimental Spectroscopy

Samples of compounds **1**–**3** were prepared as described in the literature [40,60]. Their gas phase spectra were obtained according to a procedure described in detail previously [47]. Molecules were vaporized using laser desorption and then cooled down during supersonic expansion, whereby the freezing of the vibrational degrees of freedom provided a conformational distribution corresponding to that of a sample at room temperature [46].

**Figure 8:**
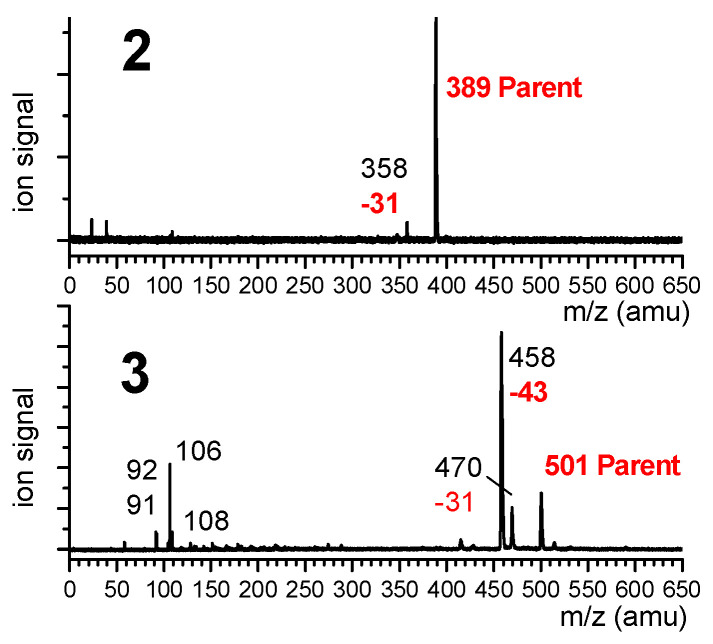
Mass spectra of **2** and **3**, recorded when tuning the UV laser on the main UV transition (band A) of these species at 37,588 cm^−1^. Parent mass species are indicated in red, as are the mass losses leading to the observed fragments.

Mass-selected UV absorption spectra of compounds **2** and **3** were recorded using the one-color resonant two-photon ionization technique (R2PI), where the ion signal was collected for the most intense peak of the mass spectrum, namely the mass channels 389 and 458 for **2** and **3**, respectively. The mass spectra (Figure 8) recorded with the UV laser tuned on the most intense UV transition emphasized that the most intense ion channel in the case of **3** was actually a fragment (*m/z* = 458) corresponding to an *m/z* loss of 43 amu, assigned to the presence of a dehydroalanine residue instead of one of the Aatc(Me) residues (presumably the result of the ejection of a Me-N=CH_2_ neutral species). The parent ion (*m/z* 501) was much less intense (23% of the 458 mass peak) and was accompanied by a weaker fragment at *m/z* 470 (17% of the 458 mass peak). Additionally, small mass peaks were observed in the 106–108 *m/z* region, which may correspond to the cleavage/rearrangement of the Cbz cap. In the case of **2**, in addition to the parent peak, the 31 amu mass channel loss was detected, although it was much less prominent (4% of the parent); the most intense feature was due to the parent peak. In the study of **1 [40]**, no such fragmentations were observed and the R2PI signal was collected on the parent channel (*m/z* = 277).

Additional investigations were needed in order to determine the origin of the fragment ions, in particular whether the process originated from photoionization of small neutrals formed during the laser desorption process or whether it occurred subsequent to the photoionization of intact, laser-desorbed molecules. The low dependence of the fragment channel *m/z* 458 for **3** with the intensity of the desorption laser suggested that fragmentation takes place within the ion. This point was confirmed by IR/UV spectroscopy (described in Section 2.1.2), which showed that both parent and fragment mass channels led to the same IR spectrum.

### 4.3. Solution-State Spectroscopy

Fourier transform infrared absorption spectra were recorded at 300 K for solutions in CHCl_3_ on an FT-IR Perkin Elmer Spectrum Two instrument. Solutions were held in a Specac Omni-Cell NaCl solution cell (1 mm path length). Spectra were recorded at three different concentrations (0.5 mM, 5 mM; 10 mM) to ensure that no concentration-related effects were occurring.

^1^H NMR spectra were recorded at 300 K for solutions in CDCl_3_ (5 mM for 1D experiments, 20 mM for 2D experiments) on a Bruker spectrometer operating at 400 MHz. Chemical shifts (δ) were measured in parts per million (ppm) with reference to the residual protonated solvent (δ = 7.26 ppm). For well-separated 1D spectral signals, assignments were made with the help of standard 2D COSY, HMBC and HSQC pulse sequences. For 2D NOESY experiments, the pulse sequence was noesygpph, collecting 2048 points in f2 and 256 points in f1; the mixing times were 200 ms for **1** and **2**; 600 ms for **3**.

## Data Availability

The original data are available upon request from the corresponding author.

## Supplementary Materials

The following supporting information can be downloaded at: https://www.mdpi.com/article/10.3390/molecules28135048/s1, Section S1: Gas phase quantum chemistry modelling; Section S2: Gas phase spectroscopy of compound **3**; Section S3: Solution phase modelling; Section S4: Cbz-(Attc)_n_-NHMe compounds in solution; Section S5: Solution phase spectroscopy of compounds **1**–**3**.
