# Supplementary material for: Length-Dependent Transition from Extended to Folded Shapes in Short Oligomers of an Azetidine-Based α-Amino Acid: The Critical Role of NH···N H-Bonds
Source: Molecules. 2023 Jun 28;28(13):5048. doi: 10.3390/molecules28135048 (PMC10343408; doi:10.3390/molecules28135048)

molecules MDPI

Supplementary Information

Length-dependent transition from extended to folded shapes in short oligomers of an azetidine-based α-amino acid: the critical role of NH···N H-bonds

Dayi Liu [1,†], Jean-Xavier Bardaud [2,†,§], Zeynab Imani [1], Sylvie Robin [1,3], Eric Gloaguen [2,§], Valérie Brenner [2,#], David J. Aitken [1,*] and Michel Mons [2,*]

1 Université Paris-Saclay, CNRS, ICMMO, 91400 Orsay, France.
2 Université Paris-Saclay, CEA, CNRS, LIDYL, 91191 Gif-sur-Yvette, France.
3 Université Paris Cité, Faculté de Pharmacie, 75006 Paris, France.
† Shared first-authorship
§ New address: Université Paris-Saclay, CNRS, ISMO, 91400 Orsay, France
# New address: CEA Paris-Saclay, Direction de la Recherche Fondamentale
* Correspondence: DJA: david.aitken@universite-paris-saclay.fr; MM: michel.mons@cea.fr

Table of contents

S1. Gas phase quantum chemistry modeling 2
S1.1 Relevant gas phase structures of compounds **1-3** 2
S1.2. Gas phase structure of the most stable helical conform. of compound **3**...7
S1.3. Temperature dependence of gas phase energetics of compound **3** 7
S2. Gas phase spectroscopy of compound **3**....8
S3. Solution phase modeling 10
S3.1 Relevant quantum chemistry conformations of compounds **1-3**....10
S3.2. Solution phase IR spectra and theoretical modeling....16
S4. Cbz-(Attc)$_n$-NHMe compounds in solution 17
S4.1. Theoretical landscapes of Cbz-(Attc)$_n$-NHMe in solution....17
S4.2. IR in solution: experiment and theoretical modeling 18
S5. Solution phase spectroscopy of compounds **1-3**....19
S5.1 IR spectroscopy dilution experiments....19
S5.2 ^{1}H NMR chemical shifts of NH signals....20
S5.3 ^{1}H NMR DMSO-d_6 titration experiments 21

S1. Gas phase quantum chemistry modeling

S1.1 Relevant gas phase structures of compounds 1-3

Listed below, in a xyz format, are the most relevant gas phase structures of compounds **1-3**, shown in Figure 2, at the RI-B97-D3 (BJ-abc)/def2-TZVPPD level of theory.

Compound **1**: conformation 5°-6γ (*g*+)

C	0.91305	-1.43128	-4.75172
O	0.97728	-2.27584	-3.87027
N	1.75634	-1.31512	-5.80898
C	2.87737	-2.16354	-6.07304
C	3.52545	-1.64583	-7.37663
O	3.07101	-0.67488	-7.98192
N	4.61056	-2.33867	-7.78279
O	-0.04364	-0.45604	-4.82438
C	-0.95634	-0.37715	-3.69098
C	5.34299	-1.98151	-8.98519
C	2.69006	-3.71339	-6.07325
N	4.01271	-3.83263	-5.40818
C	3.92615	-2.42377	-4.94618
H	1.61876	-0.57164	-6.48534
H	4.90271	-3.12143	-7.20110
H	5.75940	-0.97142	-8.90667
H	4.68872	-2.00792	-9.86260
H	6.15709	-2.69523	-9.12270
H	1.85649	-3.99984	-5.42182
H	2.61422	-4.21369	-7.04426
H	4.86579	-1.86334	-4.98918
H	3.46117	-2.32584	-3.95833
C	4.16457	-4.86261	-4.39486
H	3.38597	-4.79833	-3.61484
H	5.14598	-4.76636	-3.91863
H	4.10932	-5.85030	-4.86450
H	-1.87518	0.02763	-4.12005
H	-1.13597	-1.38635	-3.31471
C	-0.41946	0.52455	-2.61231
C	0.36355	0.00744	-1.57234
H	0.57418	-1.05702	-1.54859
C	0.87737	0.85287	-0.58923
H	1.48271	0.44209	0.21432
C	0.61389	2.22327	-0.63396
H	1.01285	2.88060	0.13373
C	-0.16760	2.74641	-1.66554
H	-0.37983	3.81133	-1.70254
C	-0.68002	1.89958	-2.64794
H	-1.28918	2.30657	-3.45169

Compound **2**: conformation 5°-6γ/5°-6γ (*g*+)

C	1.7607332	-0.4737004	-2.822154
C	2.9521208	-1.4443056	-2.9757286
O	3.2035636	-2.2977574	-2.1266008
N	3.6774352	-1.2734754	-4.1033385
C	4.8343206	-2.0987602	-4.4027996
C	2.1524612	1.0091281	-3.1121188
N	1.7245954	0.8055437	-4.5194901
C	0.9039182	-0.3661292	-4.1224146
H	3.381773	-0.5360267	-4.7384249
H	4.5518987	-3.1525912	-4.4985906
H	5.5835292	-2.0231534	-3.6078717
H	5.2720504	-1.7578188	-5.3427082
H	1.5027792	1.6980301	-2.5608641
H	3.204611	1.2846249	-2.9849635
H	0.9385823	-1.2112115	-4.8179991
H	-0.1328068	-0.103306	-3.8842606
C	1.0489441	1.9063683	-5.1848456
H	0.1814033	2.2731336	-4.6093787
H	0.702546	1.5805314	-6.1711716
H	1.7506697	2.7347014	-5.327418
C	-2.3243845	-0.1326876	1.794368
O	-2.1251295	-0.9681406	2.6631431
N	-1.5928665	0.040837	0.6648243
C	-0.4522346	-0.7361592	0.2838116
C	0.0276035	-0.1753358	-1.0748478
O	-0.5624268	0.7640994	-1.6154336
N	1.1137938	-0.7966406	-1.5833912
O	-3.349251	0.7770451	1.8128763
C	-4.1525999	0.7996224	3.0272305
C	-0.5465219	-2.2945448	0.290534
N	0.8294816	-2.3291906	0.8319885
C	0.7156306	-0.9313342	1.3012978
H	-1.8479588	0.7704203	0.0083164
H	1.5290736	-1.5691407	-1.0523124
H	-1.3064849	-2.6298205	1.006738
H	-0.6747168	-2.7973914	-0.6744197
H	1.6154289	-0.3222206	1.1604096
H	0.3480068	-0.843302	2.331253
C	1.1751216	-3.3589377	1.7906485
H	0.4937688	-3.3762688	2.660323
H	2.1967107	-3.1942715	2.1487644
H	1.1439336	-4.3376541	1.3007748
H	-5.1293907	1.1534367	2.6904701
H	-4.2386829	-0.2190675	3.4106333
C	-3.5674707	1.7261562	4.0591305
C	-2.6900592	1.2428539	5.0378158
H	-2.4397081	0.1867082	5.0480208
C	-2.1335561	2.1110574	5.9768681
H	-1.4548097	1.7260712	6.733136
C	-2.4474752	3.4711073	5.948115
H	-2.0150164	4.1461476	6.6815764
C	-3.3222142	3.961082	4.9767391
H	-3.5736246	5.0178528	4.9525309
C	-3.8775196	3.0913581	4.0388967
H	-4.5595791	3.4724269	3.2823115

Compound **3**: 5°-6γ/5°-6γ/5°-6γ (*g*+)

C	-0.0875595	0.1336363	2.7871424
N	0.0837614	-0.4353398	4.0009555
C	0.3078286	0.2679375	5.2301085
C	0.4814179	-0.7975360	6.3360651
O	0.4187834	-2.0007124	6.0885623
N	0.7016809	-0.2974304	7.5719106
C	0.8818187	-1.1595277	8.7270646
C	-0.6735742	1.4084660	5.6447492
N	0.4950123	2.1812628	6.1346550
C	1.4098525	1.3723124	5.2897246
H	0.0584127	-1.4579583	4.0716714
H	0.7335506	0.7153187	7.6622413
H	1.7491300	-1.8150897	8.5958954
H	0.0027226	-1.7933740	8.8834894
H	1.0349752	-0.5341386	9.6085074
H	-1.1456012	1.8548945	4.7622864
H	-1.4229761	1.1722228	6.4072008
H	2.3613730	1.1069638	5.7627047
H	1.5788260	1.8091118	4.2991770
C	0.4871030	3.6199936	5.9375029
H	0.3203370	3.8985418	4.8825713
H	1.4448876	4.0393042	6.2628982
H	-0.3044519	4.0682917	6.5471418
C	-0.3032637	-0.8739086	1.6353946
O	-0.0808748	1.3475804	2.5724199
C	0.7079467	-2.0631125	1.6736558
N	-0.2878185	-2.8251751	2.4574355
C	-1.3807287	-1.9514396	1.9802054
H	0.8728163	-2.4646945	0.6663033
H	1.6641558	-1.8945134	2.1817602
H	-2.1254785	-1.6891393	2.7394933
H	-1.8676035	-2.3190402	1.0690712
C	-0.3965816	-4.2534195	2.2390004
H	-0.5599305	-4.5070957	1.1763729
H	-1.2312235	-4.6489081	2.8271745
H	0.5195569	-4.7452093	2.5818608
C	-1.0671893	0.3332611	-4.3728221
O	-0.9919540	1.5258378	-4.6274803
N	-0.9501402	-0.2346156	-3.1461871
C	-0.7212364	0.4681335	-1.9202645
C	-0.6418682	-0.6053155	-0.8085063
O	-0.7577238	-1.8036200	-1.0775591
N	-0.4375692	-0.1073783	0.4298295
O	-1.3008044	-0.6459468	-5.3026075
C	-1.3167970	-0.2094091	-6.6912670
C	-1.6445121	1.6663923	-1.5390692
N	-0.4467233	2.3714809	-1.0313860
C	0.4436252	1.5035636	-1.8342506
H	-1.0093753	-1.2426929	-3.0507157
H	-0.3562632	0.9084831	0.5390347
H	-2.0728495	2.1253060	-2.4386946
H	-2.4243059	1.4842675	-0.7913774
H	1.3617723	1.1878227	-1.3266006
H	0.6727956	1.9114744	-2.8265272
C	-0.3603302	3.8062506	-1.2146841
H	-0.4806891	4.1061706	-2.2713143
H	0.6118700	4.1617739	-0.8579117
H	-1.1380614	4.2970121	-0.6203008
H	-1.9919911	-0.9131377	-7.1832125
H	-1.7348178	0.7984126	-6.7378366
C	0.0592962	-0.2651480	-7.2989381
C	0.8972805	0.8567984	-7.2740060
H	0.5385750	1.7735478	-6.8166216
C	2.1810709	0.7908528	-7.8155427
H	2.8225555	1.6675074	-7.7923016
C	2.6411407	-0.3967891	-8.3876399
H	3.6404141	-0.4466644	-8.8113917
C	1.8117883	-1.5196019	-8.4172323
H	2.1635726	-2.4454356	-8.8639928
C	0.5284837	-1.4513877	-7.8755587
H	-0.1170442	-2.3262792	-7.8986502

Compound **3**: 5°-$6\gamma^{N}$-π_{am}/5°-6γ (*g*+)

C	0.3380347	-0.4788269	3.1650906
N	-0.4129237	-1.4253931	2.5526524
C	-0.3189538	-2.8414185	2.7281355
C	-1.2866919	-3.4736081	1.7026760
O	-1.9328621	-2.7786827	0.9148913
N	-1.3565726	-4.8195277	1.7426616
C	-2.1895995	-5.5768774	0.8244105
C	1.0851128	-3.5229946	2.7113045
N	0.6138083	-4.4161927	3.8011892
C	-0.4574437	-3.4500037	4.1554503
H	-1.0113679	-1.1563230	1.7782746
H	-0.7494320	-5.2891983	2.4111291
H	-3.2207390	-5.2127848	0.8539815
H	-1.8295614	-5.4869259	-0.2067345
H	-2.1702986	-6.6273292	1.1201546
H	1.8608546	-2.8187494	3.0316701
H	1.3853075	-4.0329951	1.7903003
H	-1.4188450	-3.9022613	4.4214952
H	-0.1480907	-2.7227237	4.9150566
C	1.5652454	-4.7853872	4.8347838
H	2.0400022	-3.9059861	5.3028038
H	1.0553678	-5.3639103	5.6123725
H	2.3488428	-5.4155561	4.4009345
C	0.0689923	0.9530146	2.6708717
O	1.1252546	-0.6944764	4.0832886
C	1.2267572	1.9664260	2.8750993
N	0.2556766	2.9309472	3.4485243
C	-0.7104119	1.8419907	3.6784214
H	1.7386029	2.3027873	1.9707471
H	1.9476106	1.5822482	3.6084351
H	-0.6604957	1.4144573	4.6917208
H	-1.7527883	2.0758575	3.4277308
C	0.6774188	3.7073261	4.5962811
H	-0.1657354	4.2959299	4.9745063
H	1.0639853	3.0783022	5.4215368
H	1.4673256	4.4036767	4.2957109
C	-0.0112519	0.6133175	-3.4714992
O	-1.0221829	1.0835841	-3.9703723
N	0.2590718	0.5172328	-2.1449443
C	-0.6000144	0.9485094	-1.0856195
C	0.1574139	0.6786793	0.2308616
O	1.2879960	0.1953339	0.2452984
N	-0.5294929	1.0125791	1.3516401
O	1.0327976	0.0826692	-4.1823288
C	0.9760226	0.2678974	-5.6252761
C	-1.2129052	2.3843800	-1.1178867
N	-2.4670205	1.8130431	-0.5642401
C	-2.0797106	0.4557717	-1.0347997
H	1.1481554	0.1356184	-1.8412688
H	-1.4351181	1.4623182	1.2119012
H	-1.3188218	2.7329306	-2.1516938
H	-0.7393735	3.1515684	-0.4959720
H	-2.3191980	-0.3647555	-0.3531649
H	-2.4426979	0.2372032	-2.0459735
C	-3.7254098	2.3606415	-1.0380748
H	-3.7989209	2.3503354	-2.1395610
H	-4.5556414	1.7770068	-0.6268566
H	-3.8296030	3.3938614	-0.6901676
H	-0.0669108	0.2219060	-5.9454875
H	1.5240755	-0.5882706	-6.0247820
C	1.6224076	1.5647953	-6.0324466
C	3.0042417	1.6263175	-6.2496274
H	3.6017751	0.7238802	-6.1449778
C	3.6166319	2.8307710	-6.5945150
H	4.6895736	2.8655772	-6.7619699
C	2.8492831	3.9896864	-6.7264498
H	3.3239453	4.9290365	-6.9964378
C	1.4703008	3.9371709	-6.5138242
H	0.8696634	4.8366450	-6.6180376
C	0.8596963	2.7312867	-6.1687343
H	-0.2101649	2.6866400	-5.9902700

Compound **3**: f^{N}-$\pi_{am}{}^{N}$-10^{N}-10 (*g*+)

C	0.9241170	-0.8347466	-1.4998513
N	0.4742507	0.4262449	-1.2830241
C	0.6788007	1.4815387	-2.2567723
C	2.1504587	1.9051547	-2.4183625
O	2.5014867	2.5860109	-3.3799209
N	2.9794969	1.5589510	-1.4073703
C	4.3930073	1.8879409	-1.4471433
C	-0.1110320	1.2767470	-3.5755641
N	-1.2008023	2.1356138	-3.0433625
C	-0.2833902	2.6777479	-2.0183166
H	0.0074778	0.6595586	-0.4147990
H	2.6586245	0.9361275	-0.6772157
H	4.9056598	1.3467726	-2.2512092
H	4.5297120	2.9588932	-1.6226963
H	4.8384678	1.6138672	-0.4889098
H	-0.3997591	0.2505763	-3.8122537
H	0.4301098	1.7302148	-4.4152382
H	0.1978299	3.6177427	-2.3242417
H	-0.7049323	2.7788865	-1.0137091
C	-1.8090752	3.0821262	-3.9558467
H	-2.3650248	2.5413378	-4.7295862
H	-2.5182855	3.7171423	-3.4124631
H	-1.0667958	3.7351522	-4.4536847
C	0.6769746	-1.8467372	-0.3685285
O	1.4700949	-1.1888200	-2.5401179
C	1.6746575	-3.0324493	-0.2963856
N	0.5270790	-3.9656951	-0.1768814
C	-0.3873144	-2.9292459	-0.6904690
H	2.3641914	-3.0333008	0.5518498
H	2.2224893	-3.1258945	-1.2423960
H	-0.5600179	-2.9864543	-1.7756490
H	-1.3512879	-2.8443180	-0.1747917
C	0.5965702	-5.2189793	-0.9009358
H	-0.3536774	-5.7550534	-0.8010641
H	0.8090963	-5.0817288	-1.9785593
H	1.3847407	-5.8463112	-0.4712418
C	-1.3748894	0.9154698	2.2057357
O	-0.9411560	1.5316008	1.2431894
N	-0.6046501	0.1441684	3.0274171
C	0.8421158	0.1419088	2.8971547
C	1.3100039	-0.4371781	1.5546108
O	2.4364140	-0.2009063	1.1187212
N	0.4183832	-1.2316045	0.9194222
O	-2.6785033	0.8648397	2.5749962
C	-3.6246094	1.2470744	1.5199611
C	1.5285852	1.4611258	3.3440698
N	1.7297595	0.8728796	4.6902591
C	1.5283896	-0.4801004	4.1433059
H	-1.0227913	-0.2029525	3.8773948
H	-0.4829933	-1.3761967	1.3494306
H	0.9105891	2.3620419	3.3155086
H	2.4630763	1.6165342	2.7888765
H	2.4633724	-0.9839668	3.8536681
H	0.9239614	-1.1574846	4.7600503
C	2.9813802	1.1470310	5.3675859
H	3.0297073	0.5738523	6.2994217
H	3.8649741	0.8918360	4.7518684
H	3.0326653	2.2107420	5.6214826
H	-3.3284365	2.2143807	1.1103412
H	-4.5688545	1.3462110	2.0586623
C	-3.6917745	0.1907203	0.4505702
C	-4.2496201	-1.0638635	0.7357528
H	-4.6529282	-1.2588537	1.7266302
C	-4.2900455	-2.0574575	-0.2409999
H	-4.7276616	-3.0250902	-0.0117684
C	-3.7744695	-1.8054847	-1.5159411
H	-3.8052453	-2.5806875	-2.2765915
C	-3.2184837	-0.5600546	-1.8063657
H	-2.8033375	-0.3460749	-2.7860112
C	-3.1771339	0.4323891	-0.8271267
H	-2.7297923	1.3885374	-1.0745711

S1.2. Gas phase structure of the most stable helical conformation of compound 3

The most stable helical conformation of compound **3**, the f^{N}-π_{am}^{N}-10^{N}-10 (*g*+) structure, corresponds to an incipient 3_{10}-helix and features three successive δ motifs (δ-δ-δ). The structural analysis reveals, in addition to the two parallel C10 H-bonds, the occurrence of extra interactions between the Cbz phenyl ring and i) a β2Ha atom of the second residue side chain and ii) the lone pair of the third residue azetidine N atom, as revealed by the short relevant interatomic distances (< 300 pm) shown in Figure S1.2.

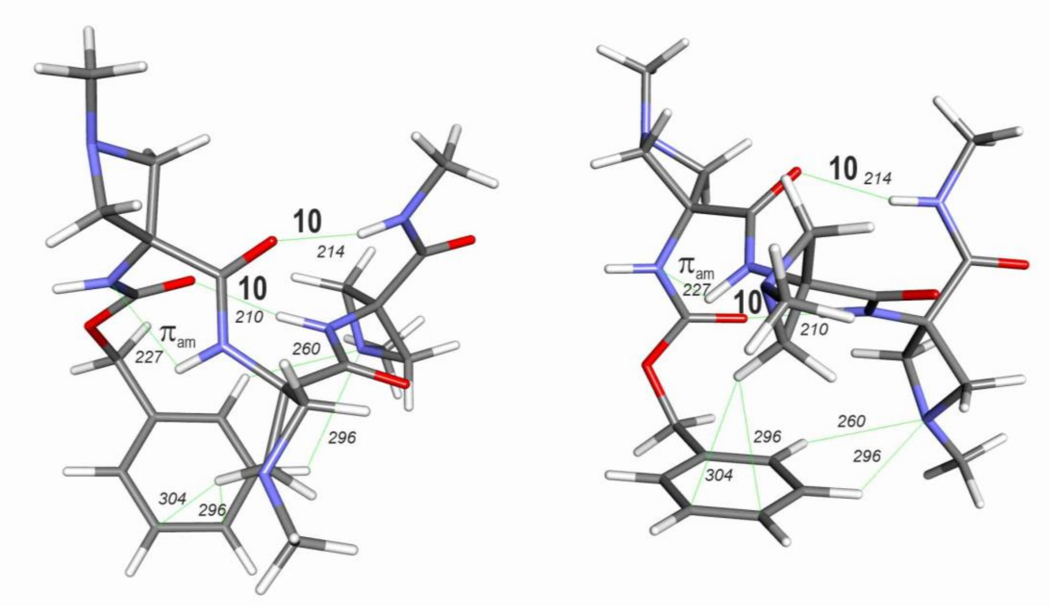

Figure S1.2. Two views of the quantum chemistry structure (RI-B97-D3 (BJ-abc)/def2-TZVPPD level) of the f^{N}-π_{am}^{N}-10^{N}-10 (*g*+) conformation of compound **3**, revealing the short dispersive interactions taking place between the Cbz moiety and the side chains of the second and third residues. The relevant interatomic distances are given in pm.

S1.3. Temperature dependence of gas phase energetics of compound 3

The phenomenon of kinetic trapping (Figure S1.3) which can occur in a supersonic expansion, stems from the coexistence, in a given conformational population, of entropically favored (basin 1) and disfavored (basin 2) species, whose conformational basins are separated by high interconversion barriers, which can only be crossed during the early expansion, characterized by a high conformational temperature inherited from the vaporization process, whether thermal or by laser-desorption.

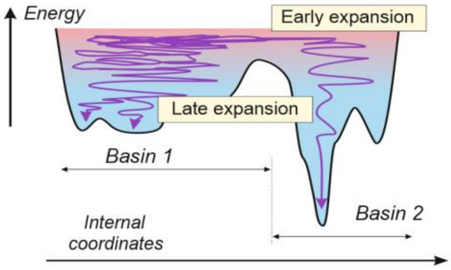

Figure S1.3. Sketch of the conformational population taking place in a supersonic expansion illustrating the phenomenon of kinetic trapping. The sudden drop in the interconversion rate as expansion develops leads to the trapping of the early conformational populations.

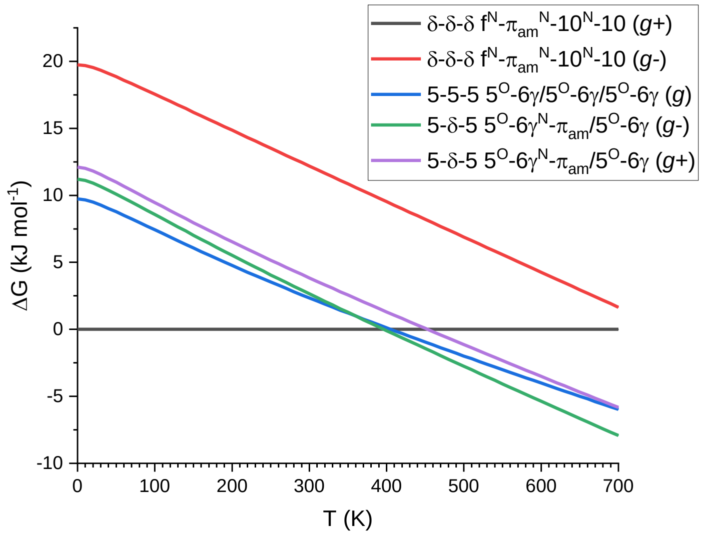

Figure S1.4. Temperature dependence of the stability of the most stable conformations of compound **3**, relative to the most stable helical form.

The analysis of the temperature dependence of the stability of the most stable conformations of compound **3** (Figure S1.4) shows that this species is fairly described by the sketch of Figure S1.3.

At high temperature, the compact helical f^N-$\pi_{am}{}^N$-10^N-10 (*g*+) conformation is less stable than its competitors. The reverse is true at low temperature. However, due to the high barrier between helical δ-δ-δ backbones and extended (5-5-5) or semi-extended (5-δ-5) forms (typically illustrated by the two basins in Figure S1.3), interconversions are rapidly blocked in the expansion, freezing the conformation populations of each basin to that of the early expansion. Notwithstanding, further interconversion within each basin eventually provides additional conformational relaxation, leading *in fine* to a limited number of populated conformations. Such a kinetic trapping phenomenon hampers the most stable structure f^N-$\pi_{am}{}^N$-10^N-10 (*g*+) structure at low temperature but poorly populated in the early expansion to eventually funnel the population in the late expansion.

S2. Gas phase spectroscopy of compound 3

The gas phase IR spectroscopy of compound **3** was challenging for the IR/UV double resonance technique, because of the fragmentation processes taking place in the ion, and the observation of hybrid IR spectra.

A first IR/UV spectrum was obtained from the major UV band A (at 37588 cm^{-1}), by collecting the *m*/*z* = 458 (Figure S2.1, top). It exhibited 3 features, the most red-shifted band exhibiting a prominent shoulder, suggesting 4 NH stretch bands as expected from the four NH stretches of the molecule. It was checked that the *m*/*z* 501 parent channel exhibited the same IR depletions as those recorded on the *m*/*z* 458 fragment channel, demonstrating that this fragmentation takes place after photoionization of **3**, rather than in neutral fragments formed during the laser desorption process.

In contrast, the IR/UV spectrum obtained from the UV band X (at 37586 cm^{-1}), by collecting the *m*/*z* = 458 signal (Figure S2.1, center), exhibited 5 NH stretch bands, some of them being broad or doublet-like; among them four overlapped with IR bands obtained from the UV band A. Additionally, the maximum depopulation in this case was less than 50 %, in contrast to that obtained when probing on the A band. This suggested that, when using the X band with the UV laser, a mixture of two conformers was actually being probed, namely conformer A and a second one, which was labelled B. By subtracting the

(weighted) IR spectrum of A from that obtained from band X, the IR spectrum of B was obtained; it comprised four absorption IR bands as expected (Figure S2.1, bottom). The weight needed (0.60) suggested that B represents at best 40 % of the population probed at the UV band X, supporting the fact that i) B is a minor conformer (its population is much less than 40 %, taking into account the integrated UV line shapes assignable to B and A respectively) and ii) in addition to the major A band, conformer A also presents a minor UV absorption feature at 37586 cm^{-1}, which might be a hot band resulting from insufficient cooling in the supersonic expansion for this large molecule. Finally, as for conformer A, it was found that the parent mass channel, when exciting the UV band X (at 37586 cm^{-1}), exhibited the same depopulations as the *m*/*z* 458 mass channel, demonstrating that this UV band is due to **3** and not to a neutral formed in the laser-desorption process.

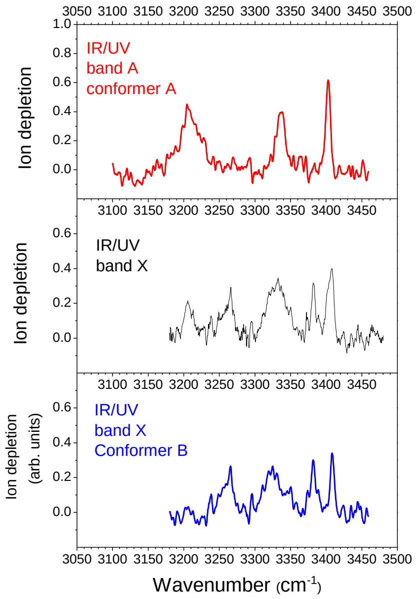

Figure S2.1. (*top and center*) IR/UV spectrum obtained by tuning the UV laser on the UV bands A and X, respectively, giving rise to the IR spectrum of conformer A (*top*) and to a hybrid spectrum (*center*), comprising contributions of A and of a second conformer labelled B; (*bottom*) Reconstructed IR spectrum of conformer B, by subtraction of a weighted contribution of A to the spectrum obtained from band X.

S3. Solution phase modeling

S3.1 Relevant quantum chemistry conformations of compounds 1-3

Listed below, in a xyz format, are the most relevant solution phase structures of compounds **1**-**3**, at the RI-B97-D3 (BJ-abc)/def2-TZVPPD + COSMO level of theory.

Compound **1**: conformation 5°-6γ (g+)

C	-1.2600196	-0.1401942	0.1044200
O	-1.2080377	-0.9975321	-0.7676062
N	-0.4088006	-0.0109919	1.1510111
C	0.7160394	-0.8549079	1.4288465
C	1.3659217	-0.3185378	2.7227118
O	0.9047295	0.6637921	3.3177542
N	2.4501006	-0.9948263	3.1370164
O	-2.2141853	0.8391142	0.1699792
C	-3.1281153	0.9270403	-0.9671252
C	3.1863132	-0.6287130	4.3364963
C	0.5299436	-2.4046476	1.4539835
N	1.8461866	-2.5340472	0.7782249
C	1.7618664	-1.1294935	0.3032324
H	-0.5479070	0.7450861	1.8121987
H	2.7462131	-1.7878745	2.5717687
H	3.5803950	0.3897951	4.2572712
H	2.5422878	-0.6792036	5.2204197
H	4.0162887	-1.3257011	4.4573468
H	-0.3097307	-2.7034399	0.8160022
H	0.4616216	-2.8893861	2.4327224
H	2.7015516	-0.5697977	0.3376178
H	1.2963240	-1.0405621	-0.6852540
C	1.9843711	-3.5740542	-0.2284625
H	1.1978842	-3.5117450	-1.0009305
H	2.9606945	-3.4842787	-0.7157780
H	1.9278552	-4.5572354	0.2498971
H	-4.0480241	1.3254442	-0.5362913
H	-3.3052807	-0.0780444	-1.3534783
C	-2.5849861	1.8425264	-2.0306792
C	-1.7864151	1.3422029	-3.0678357
H	-1.5717551	0.2788450	-3.1069124
C	-1.2642028	2.2019267	-4.0351182
H	-0.6469717	1.8038971	-4.8360331
C	-1.5361844	3.5711762	-3.9770878
H	-1.1311861	4.2398511	-4.7317378
C	-2.3349907	4.0776694	-2.9491961
H	-2.5548788	5.1407150	-2.9030061
C	-2.8553077	3.2161181	-1.9824655
H	-3.4796087	3.6100143	-1.1839232

Compound **2**: conformation f^{N}-π_{am}^{N}-10 (*g*+)

C	1.0902569	-0.1040711	-2.2579089
N	0.6506365	1.1308624	-1.9616388
C	0.6763305	2.2089438	-2.9343053
H	0.2846739	1.3349881	-1.0392516
C	0.9380458	-1.1678272	-1.1568892
O	1.5393733	-0.4158412	-3.3666357
C	1.9849954	-2.3131593	-1.1631591
N	0.8841400	-3.3074540	-1.0772391
C	-0.0913414	-2.2799265	-1.4988071
H	2.6893836	-2.3288272	-0.3290072
H	2.5191783	-2.3410844	-2.1205859
H	-0.3053317	-2.2939393	-2.5764089
H	-1.0331712	-2.2655407	-0.9395226
C	0.9941159	-4.4927659	-1.9094483
H	0.0696874	-5.0762035	-1.8426600
H	1.1774034	-4.2485402	-2.9729452
H	1.8194855	-5.1170115	-1.5511921
C	-1.0238343	1.5908186	1.5022846
O	-0.5386073	2.2515667	0.5902988
N	-0.3150109	0.7501031	2.2988875
C	1.1303284	0.6564508	2.2053714
C	1.6082356	0.0939481	0.8569974
O	2.7619504	0.2633020	0.4681398
N	0.6866593	-0.6141497	0.1595156
O	-2.3422236	1.5753940	1.8287377
C	-3.2426076	2.0416543	0.7693413
C	1.8845732	1.9152252	2.7128824
N	2.0319801	1.2728617	4.0438134
C	1.7358491	-0.0475906	3.4498276
H	-0.7957329	0.2829162	3.0533016
H	-0.2381861	-0.7134773	0.5516144
H	1.3215429	2.8516349	2.7079259
H	2.8372389	2.0347170	2.1822439
H	2.6365025	-0.6158153	3.1750757
H	1.0612985	-0.6875507	4.0301192
C	3.3111335	1.4256486	4.7148809
H	3.3237182	0.8205394	5.6273712
H	4.1619583	1.1183821	4.0781823
H	3.4515199	2.4740597	4.9975022
H	-2.9250203	3.0313432	0.4369249
H	-4.2059512	2.1155906	1.2764143
C	-3.2816330	1.0590451	-0.3702584
C	-3.7968227	-0.2296464	-0.1692665
H	-4.1862529	-0.5054197	0.8075651
C	-3.8073234	-1.1568602	-1.2104984
H	-4.2063965	-2.1536120	-1.0446057
C	-3.3079671	-0.8028325	-2.4674983
H	-3.3117060	-1.5260234	-3.2779505
C	-2.7980757	0.4787172	-2.6760923
H	-2.4000022	0.7545262	-3.6480873
C	-2.7833624	1.4033462	-1.6306458
H	-2.3705014	2.3952382	-1.7867056
H	0.3128323	3.1164306	-2.4507990
H	0.0385030	1.9780544	-3.7945355
H	1.6935312	2.3788614	-3.3006708

Compound **2**: conformation 5°-$6\gamma^{N}$-π_{am} (*g*-)

C	3.6125995	0.4416333	1.8095850
N	2.6758501	1.3076495	2.2460717
C	2.6202933	1.7660827	3.6254319
H	1.9318849	1.5579042	1.6140283
C	3.5324790	0.0699838	0.3217541
O	4.5271470	0.0218367	2.5229412
C	4.1906059	-1.2754854	-0.0859527
N	4.8950520	-0.5798334	-1.1934448
C	4.6399399	0.7252718	-0.5495404
H	3.5124048	-2.0735380	-0.3954371
H	4.8661024	-1.6292477	0.7030465
H	5.4739688	1.0741252	0.0765925
H	4.3244709	1.5296867	-1.2239998
C	6.2816357	-0.9412865	-1.4285784
H	6.7107944	-0.2803037	-2.1894161
H	6.8993973	-0.8695037	-0.5137347
H	6.3333070	-1.9697072	-1.8008174
C	-2.3870858	-1.2124056	-0.7991728
O	-2.8775721	-0.5299886	-1.6875659
N	-1.1193641	-1.1217504	-0.3246549
C	-0.1005332	-0.2411161	-0.8099898
C	1.1627863	-0.5153208	0.0331135
O	1.1875396	-1.3878211	0.9036564
N	2.2187185	0.2776880	-0.2600336
O	-3.0481935	-2.2076436	-0.1324336
C	-4.4892018	-2.2792292	-0.3559145
C	0.2270147	-0.1899614	-2.3360537
N	0.4071900	1.2775444	-2.2017135
C	-0.4001642	1.2831218	-0.9564738
H	-0.8280229	-1.7438926	0.4212072
H	2.0985906	0.9173163	-1.0475251
H	-0.6538951	-0.4623642	-2.9295098
H	1.1088830	-0.7363306	-2.6851645
H	-0.0381618	1.9533899	-0.1693045
H	-1.4685718	1.4495987	-1.1354396
C	-0.0321062	2.1195793	-3.3018035
H	-1.0824107	1.9271532	-3.5811191
H	0.0670289	3.1722544	-3.0181802
H	0.6015142	1.9378244	-4.1756657
H	-4.7363788	-3.3132466	-0.1081997
H	-4.6980963	-2.0933632	-1.4107413
C	-5.2245839	-1.3110494	0.5315849
C	-5.7885028	-0.1426854	0.0072057
H	-5.6996729	0.0606979	-1.0558947
C	-6.4513373	0.7601459	0.8414275
H	-6.8873766	1.6632356	0.4232800
C	-6.5506702	0.5042404	2.2104999
H	-7.0647174	1.2067938	2.8606871
C	-5.9897890	-0.6604714	2.7425821
H	-6.0686781	-0.8661445	3.8065784
C	-5.3337011	-1.5629870	1.9060237
H	-4.9003317	-2.4701958	2.3199973
H	1.8246731	2.5070937	3.7134922
H	3.5719254	2.2208881	3.9147551
H	2.4153223	0.9341337	4.3079330

Compound **2**: conformation 5°-6γ/5°-6γ (*g*+)

C	1.7579573	-0.4611756	-2.8207615
C	2.9451208	-1.4362619	-2.9699755
O	3.1876496	-2.2917596	-2.1110664
N	3.6738910	-1.2768222	-4.0888588
C	4.8307128	-2.1045675	-4.3902552
C	2.1565864	1.0191415	-3.1122532
N	1.7228059	0.8186764	-4.5183937
C	0.9037948	-0.3546935	-4.1225119
H	3.3883334	-0.5406079	-4.7296999
H	4.5469971	-3.1588700	-4.4726445
H	5.5894263	-2.0146528	-3.6059715
H	5.2543887	-1.7721522	-5.3386686
H	1.5138177	1.7127824	-2.5590389
H	3.2095958	1.2892661	-2.9864461
H	0.9394030	-1.2001946	-4.8164680
H	-0.1344767	-0.0945182	-3.8882116
C	1.0420504	1.9219400	-5.1767747
H	0.1767707	2.2842955	-4.5945507
H	0.6897400	1.5987759	-6.1616772
H	1.7413484	2.7523679	-5.3176316
C	-2.3255999	-0.1554234	1.8105823
O	-2.1306445	-0.9966007	2.6773711
N	-1.5872511	0.0302585	0.6899082
C	-0.4475647	-0.7429367	0.2918724
C	0.0244690	-0.1660637	-1.0641995
O	-0.5723972	0.7790946	-1.5931346
N	1.1069159	-0.7800761	-1.5807110
O	-3.3553458	0.7487744	1.8288935
C	-4.1609733	0.7827719	3.0465419
C	-0.5404789	-2.3008653	0.2776687
N	0.8402414	-2.3416620	0.8103987
C	0.7269718	-0.9487409	1.2983802
H	-1.8492381	0.7645191	0.0413465
H	1.5177028	-1.5555849	-1.0503198
H	-1.2940165	-2.6495269	0.9940700
H	-0.6772862	-2.7899509	-0.6923762
H	1.6232846	-0.3348557	1.1613635
H	0.3679490	-0.8764772	2.3325178
C	1.1874489	-3.3817700	1.7590338
H	0.5073579	-3.4053730	2.6296115
H	2.2088664	-3.2202328	2.1188382
H	1.1512478	-4.3562828	1.2613227
H	-5.1371871	1.1316667	2.7054307
H	-4.2469258	-0.2310535	3.4407744
C	-3.5718761	1.7232405	4.0630467
C	-2.7102244	1.2508483	5.0613767
H	-2.4777889	0.1912567	5.1040876
C	-2.1478953	2.1328989	5.9853045
H	-1.4820079	1.7560688	6.7569550
C	-2.4411295	3.4972406	5.9204061
H	-2.0046556	4.1834694	6.6409986
C	-3.3015907	3.9766047	4.9296509
H	-3.5376730	5.0359702	4.8785546
C	-3.8629976	3.0925742	4.0076532
H	-4.5356203	3.4652496	3.2386405

Compound **3**: f^N-$\pi_{am}{}^N$-10^N-10 (*g*+)

C	0.9268573	-0.8316267	-1.4754473
N	0.4612320	0.4218692	-1.2678330
C	0.6738438	1.4769036	-2.2402173
C	2.1488627	1.8872690	-2.3836502
O	2.5298981	2.5356291	-3.3642720
N	2.9539391	1.5739867	-1.3498045
C	4.3671513	1.9160137	-1.3490878
C	-0.1143042	1.2773643	-3.5618095
N	-1.1940880	2.1481509	-3.0363179
C	-0.2814982	2.6808788	-2.0041887
H	-0.0252370	0.6523290	-0.4077334
H	2.6131350	0.9786517	-0.6038176
H	4.8988293	1.4037398	-2.1587652
H	4.5026571	2.9937973	-1.4798938
H	4.7935540	1.6116077	-0.3920933
H	-0.4160740	0.2539346	-3.7952901
H	0.4293623	1.7195888	-4.4064550
H	0.2056434	3.6215182	-2.2992535
H	-0.7090460	2.7789305	-1.0025353
C	-1.7932522	3.0983335	-3.9514381
H	-2.3505388	2.5606534	-4.7260633
H	-2.4965904	3.7400403	-3.4091604
H	-1.0433931	3.7444255	-4.4476191
C	0.6641640	-1.8494327	-0.3557946
O	1.5034742	-1.1707611	-2.5093439
C	1.6425764	-3.0512671	-0.2847225
N	0.4801306	-3.9703608	-0.1783374
C	-0.4177796	-2.9125496	-0.6857756
H	2.3269040	-3.0676872	0.5672708
H	2.1957525	-3.1558932	-1.2259850
H	-0.5933043	-2.9659772	-1.7697965
H	-1.3782869	-2.8122426	-0.1686359
C	0.5377257	-5.2107676	-0.9318486
H	-0.4208869	-5.7342609	-0.8515588
H	0.7583579	-5.0469921	-2.0033860
H	1.3163304	-5.8568798	-0.5132377
C	-1.3644452	0.9221302	2.1955392
O	-0.9271971	1.5427704	1.2323597
N	-0.5931658	0.1754771	3.0292678
C	0.8507074	0.1584777	2.8938378
C	1.3069324	-0.4373104	1.5563902
O	2.4306756	-0.2073511	1.1011679
N	0.4107162	-1.2313405	0.9311380
O	-2.6713027	0.8597551	2.5482573
C	-3.6167729	1.2306362	1.4864099
C	1.5496333	1.4797046	3.3163667
N	1.7702052	0.9099690	4.6691890
C	1.5380965	-0.4515243	4.1455675
H	-1.0248829	-0.2574580	3.8321855
H	-0.4823648	-1.3890897	1.3749327
H	0.9359025	2.3830486	3.2844316
H	2.4785703	1.6253971	2.7501768
H	2.4632028	-0.9752672	3.8631227
H	0.9239301	-1.1045361	4.7757089
C	3.0519558	1.1700079	5.3004456
H	3.1217769	0.6069265	6.2368487
H	3.9057302	0.8877931	4.6562403
H	3.1330064	2.2362848	5.5349793
H	-3.3233703	2.1947652	1.0687028
H	-4.5624738	1.3316289	2.0210951
C	-3.6787530	0.1627647	0.4279978
C	-4.2262364	-1.0935082	0.7270218
H	-4.6205847	-1.2841200	1.7221777
C	-4.2683524	-2.0950985	-0.2424803
H	-4.6952520	-3.0646940	-0.0016208
C	-3.7653406	-1.8496517	-1.5241311
H	-3.7949662	-2.6313619	-2.2780976
C	-3.2198789	-0.6020166	-1.8282110
H	-2.8157676	-0.3981411	-2.8148686
C	-3.1765059	0.3984705	-0.8561942
H	-2.7395299	1.3575448	-1.1120567

Compound **3**: 5°-$6\gamma^{N}$-π_{am}/5°-6γ (*g*+)

C	0.3954439	-0.4761050	3.1197104
N	-0.3752126	-1.4193154	2.5356067
C	-0.3090802	-2.8387195	2.7233243
C	-1.3292777	-3.4562227	1.7411058
O	-1.9904511	-2.7436688	0.9731206
N	-1.4313171	-4.7935750	1.7888763
C	-2.3262070	-5.5422654	0.9201139
C	1.0763713	-3.5539364	2.6648941
N	0.6272824	-4.4193445	3.7855184
C	-0.4149941	-3.4296834	4.1609394
H	-1.0185453	-1.1431029	1.7999959
H	-0.8212305	-5.2795908	2.4418286
H	-3.3543115	-5.1826756	1.0241931
H	-2.0316427	-5.4415631	-0.1300495
H	-2.2831783	-6.5941018	1.2050792
H	1.8812169	-2.8664203	2.9471611
H	1.3305576	-4.0829045	1.7411628
H	-1.3761418	-3.8594777	4.4593982
H	-0.0722679	-2.7020420	4.9060912
C	1.6090435	-4.7868761	4.7924567
H	2.1150859	-3.9065969	5.2254619
H	1.1165737	-5.3391687	5.5994061
H	2.3660737	-5.4379628	4.3430300
C	0.0949689	0.9590399	2.6535113
O	1.2272068	-0.6957373	4.0015120
C	1.2198207	2.0026799	2.8836250
N	0.2214982	2.9238953	3.4851018
C	-0.7192904	1.8001981	3.6741198
H	1.7194470	2.3807010	1.9888536
H	1.9558068	1.6284003	3.6064039
H	-0.6699154	1.3530243	4.6778968
H	-1.7635930	2.0096743	3.4159275
C	0.6255260	3.6585806	4.6703233
H	-0.2308925	4.2108359	5.0717787
H	1.0207695	2.9985453	5.4656489
H	1.4037734	4.3809459	4.4036474
C	0.0174402	0.5991183	-3.4903139
O	-1.0023717	1.0460434	-3.9964664
N	0.2905090	0.5273622	-2.1642506
C	-0.5702902	0.9564529	-1.1028758
C	0.1908880	0.7031437	0.2140688
O	1.3326939	0.2371581	0.2307563
N	-0.4978035	1.0265483	1.3318798
O	1.0668003	0.0737522	-4.1958557
C	1.0217497	0.2517015	-5.6448042
C	-1.1974351	2.3859233	-1.1401353
N	-2.4436578	1.8046677	-0.5798273
C	-2.0449039	0.4496446	-1.0435544
H	1.1875686	0.1620638	-1.8650360
H	-1.4159727	1.4530163	1.1952276
H	-1.3102952	2.7299660	-2.1747910
H	-0.7285067	3.1612642	-0.5259474
H	-2.2734415	-0.3671801	-0.3539811
H	-2.4117590	0.2180340	-2.0501991
C	-3.7107678	2.3378674	-1.0504417
H	-3.7880317	2.3204090	-2.1515501
H	-4.5327048	1.7475044	-0.6329000
H	-3.8216218	3.3720644	-0.7085967
H	-0.0145154	0.1755022	-5.9785393
H	1.5967763	-0.5919868	-6.0307176
C	1.6405702	1.5637099	-6.0448421
C	3.0309528	1.6736955	-6.1787291
H	3.6547422	0.7975962	-6.0185267
C	3.6176669	2.8940522	-6.5149099
H	4.6969223	2.9668747	-6.6173839
C	2.8162864	4.0195182	-6.7243372
H	3.2712242	4.9702178	-6.9885338
C	1.4288193	3.9176582	-6.5976464
H	0.8019848	4.7895626	-6.7634690
C	0.8444687	2.6958862	-6.2590589
H	-0.2329020	2.6157230	-6.1504870

S3.2. Solution phase IR spectra and theoretical modeling

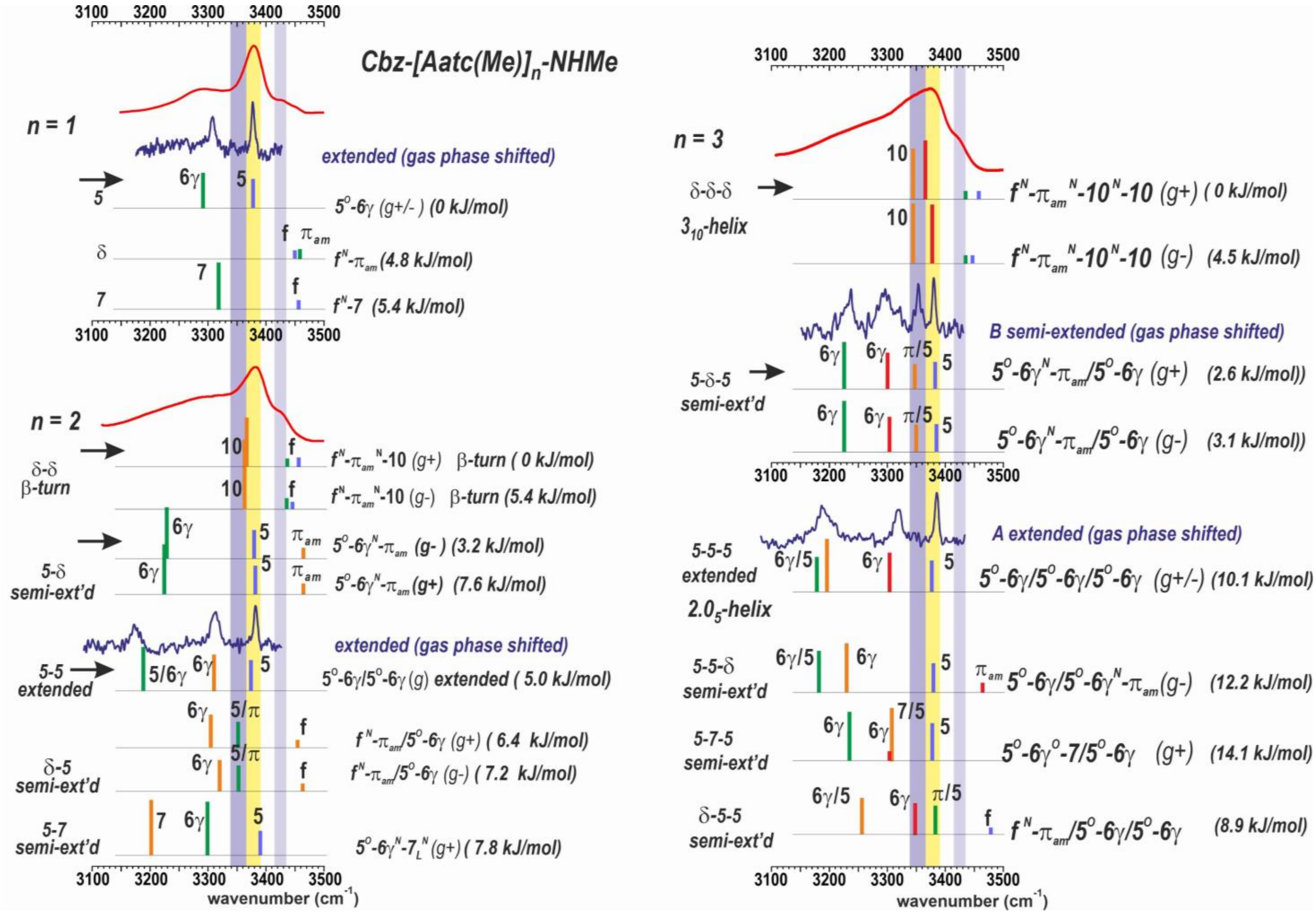

Figure S3.1. Solution state IR spectra (red traces) of compounds **1**-**3** (5 mM in chloroform) and calculated IR spectra (colored vertical bars) of stable conformations of each relevant conformational family (at 300 K; energetics in kJ·mol^{-1} between parentheses). The NH stretches are localized in each residue and the residue position from N to C terminus is rainbow color-coded from blue to red. The colored spectral regions indicate the typical locations of diagnostic spectral features: nearly free (π_{am}) NH in a δ conformation (lilac), NH engaged in a C5 H-bond (yellow) or in a C10 H-bond (mauve). The spectrum of **1** is adapted from Ref. [40]. Experimental gas phase spectra of isolated conformations are also reproduced (blue traces); a fair match with their solution counterparts is found, providing a 25 cm^{-1} red-shift is applied, which therefore constitutes the major spectroscopic effect of solvation. The arrows indicate the conformations that contribute to the solution spectrum.

The comparison between experimental IR absorption spectra and quantum chemistry solution modeling (Figure S3.1) provides interesting observations:

- i) the bands of H-bonded NHs are fairly well reproduced by theory.
- ii) in contrast, the free or nearly free NHs (π_{am}) bands are more challenging since overestimated by theory by typically 25 cm^{-1}.
- iii) the experimental gas phase spectra observed, once red-shifted by 25 cm^{-1}, are found to fairly match the IR spectra predicted in solution for these conformations, suggesting that solution leads mainly to a red-shift of ca. 25 cm^{-1} compared to its isolated counterpart.

S4. Cbz-(Attc)n-NHMe compounds in solution

S4.1. Theoretical landscapes of Cbz-(Attc)n-NHMe in solution

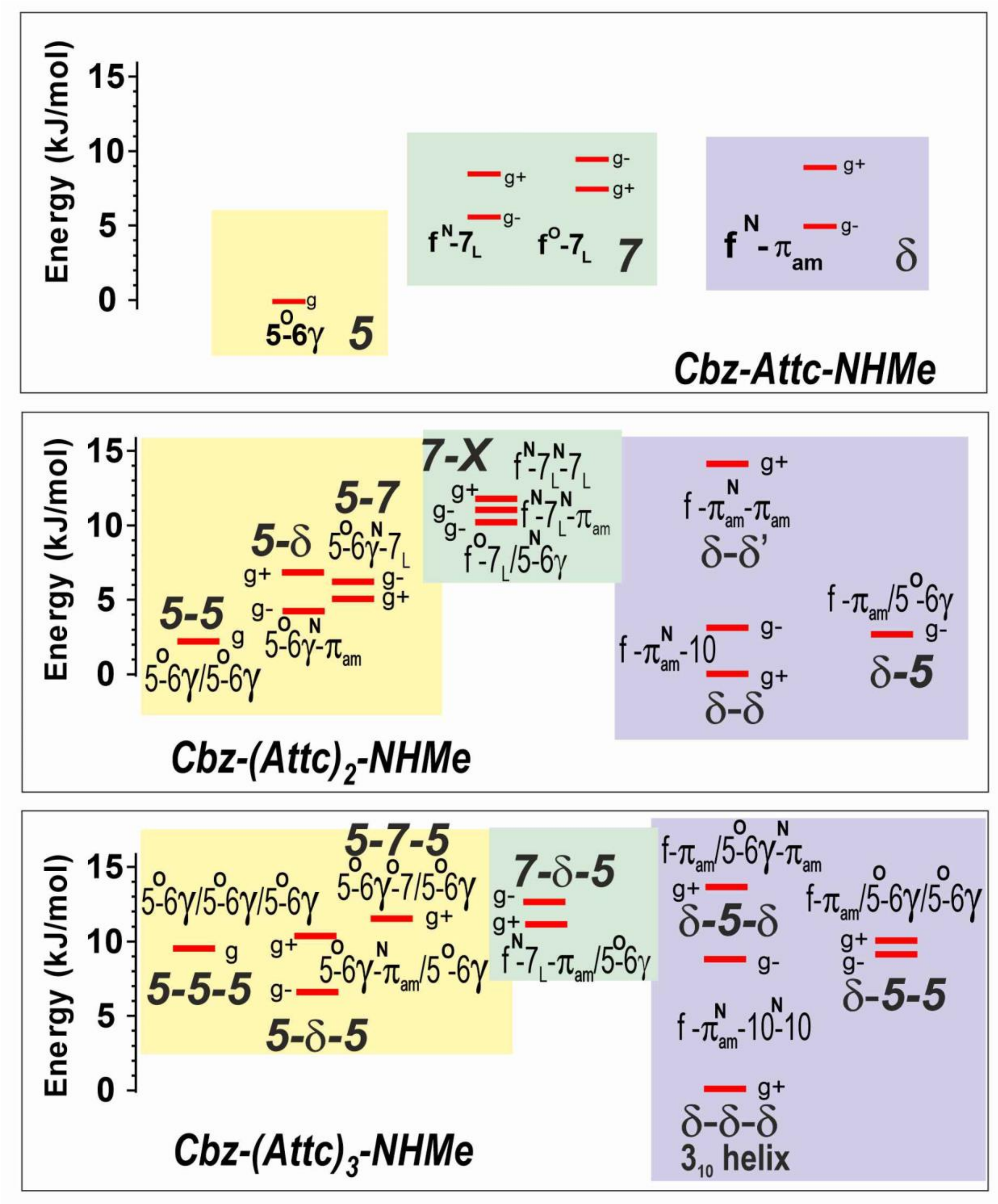

Figure S4.1. Theoretical landscapes of Cbz-(Attc)n-NHMe compounds in a chloroform solution at 300 K as obtained at the DFT-D level of theory with a solution modelling by a polarizable continuum (RI-B97D3(BJ-abc)/TZVPPD + COSMO model). Same detail captions as Figure 4. Here the absence of superscript on the first residues indicates a planar heterocyclic ring found at this level of theory.

S4.2. IR in solution: experiment and theoretical modeling

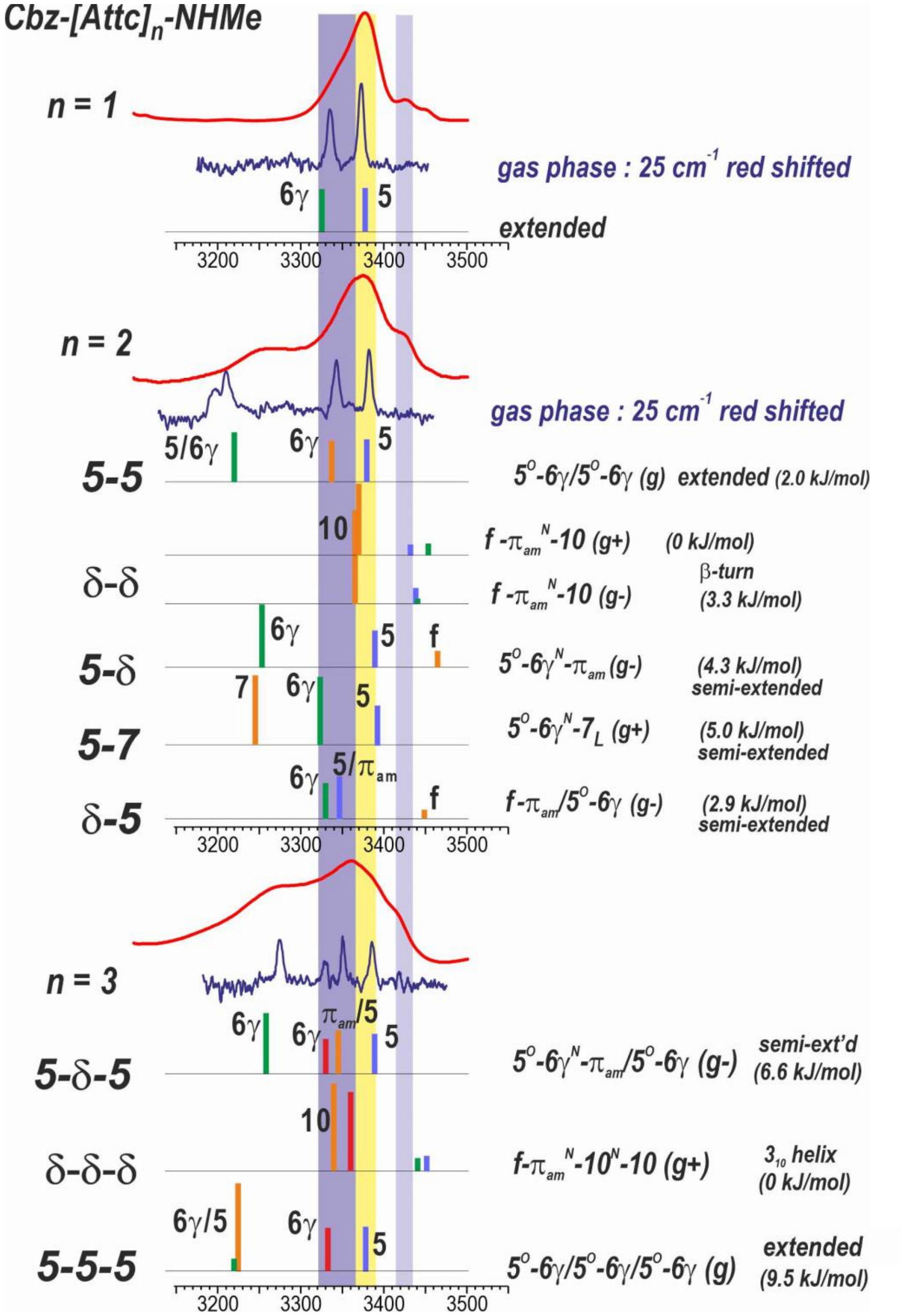

Figure S4.2. Solution state IR spectra of Cbz-(Attc)$_n$-NHMe derivatives (n = 1-3), the counterparts of compounds **1-3** (5 mM in chloroform, adapted from Ref. [36]) and the calculated IR spectra of their most energetically relevant conformations at the same level of theory. The colored zones indicate the typical locations of diagnostic spectral features: nearly free (π_{am}) NH in a δ conformation (lilac), NH engaged in a C5 H-bond (yellow) or in a C10 H-bond (mauve).

S5. Solution phase spectroscopy of compounds 1-3

S5.1 IR spectroscopy dilution experiments

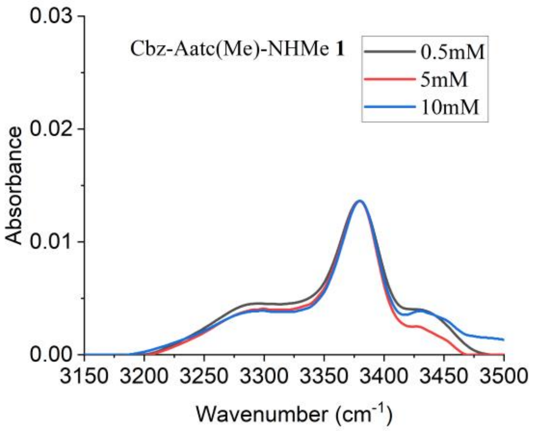

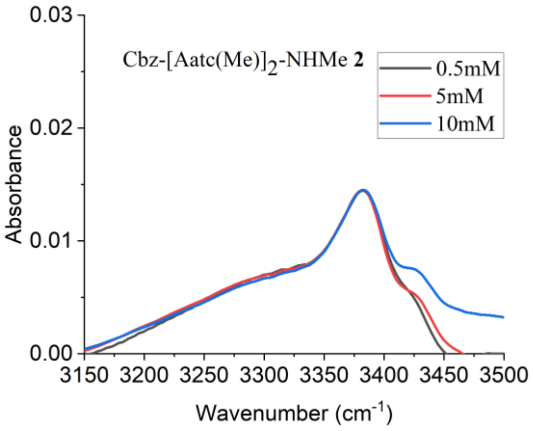

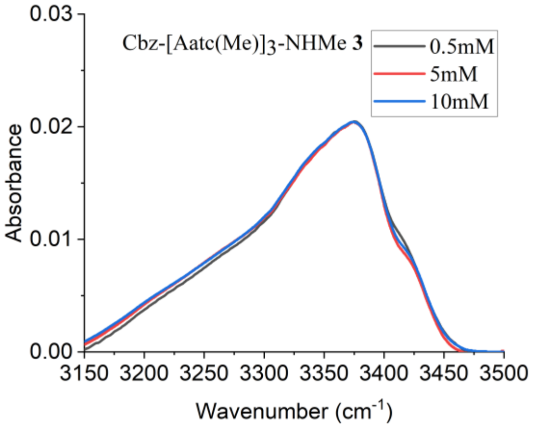

Figure S5.1. Infrared spectra of **1-3** were recorded in chloroform at three different concentrations (0.5 mM, 5 mM, and 10 mM). For each compound, the concentration is indicated by color (black, red, and blue, respectively). No significant concentration effects were noted, indicating that non-covalent interactions (hydrogen bonds) were intramolecular in nature.

S5.2 ^{1}H NMR chemical shifts of NH signals

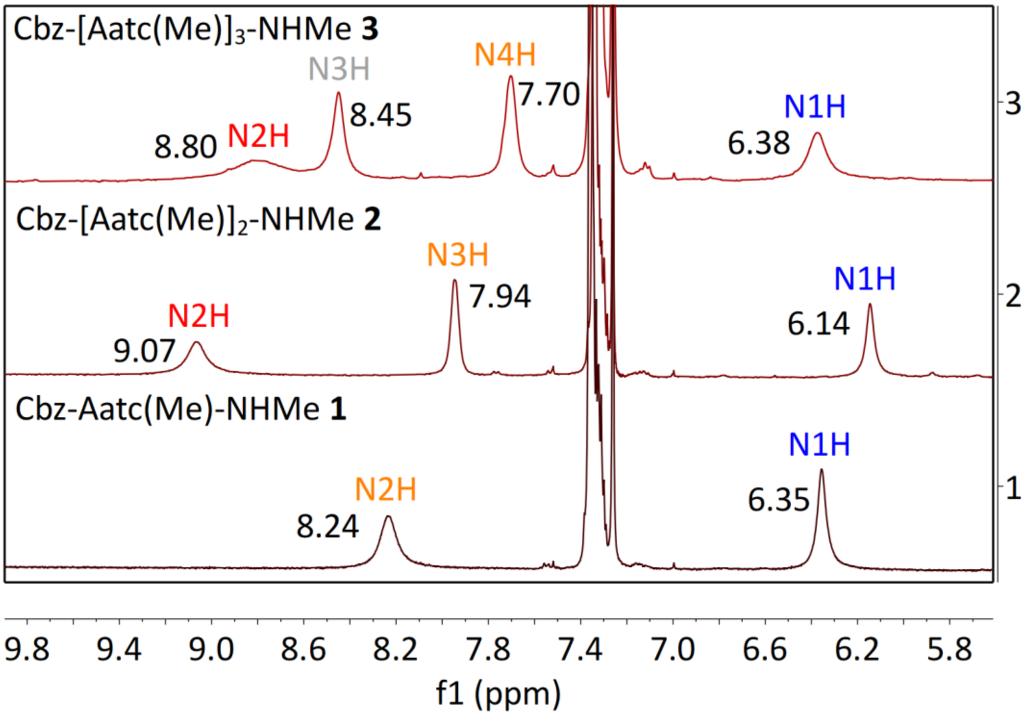

Figure S5.2. ^{1}H NMR spectra (300 K, 20 mM solutions in $CDCl_3$) of compounds **1-3** in the spectral window where the NH proton signals are located.

S5.3 ^{1}H NMR DMSO-*d*₆ titration experiments

^{1}H spectra were recorded at 300 K. Starting samples were prepared in $CDCl_3$ (400 μL) to give solutions at a concentration of 5 mM. Aliquots of DMSO-d_6 (6 × 2 μL, 2 × 4 μL, 2 × 10 μL) were added successively to the NMR tube followed, after each addition, by rapid agitation then re-recording of the ^{1}H NMR spectra.

Cbz-Aatc(Me)-NHMe **1**

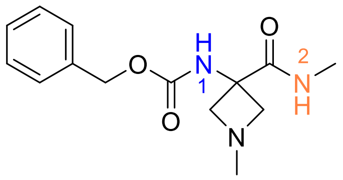

	**DMSO-*d*₆ (%, v/v)**											
	0	*0.5*	*1*	*1.5*	*2*	*2.5*	*3*	*4*	*5*	*7.5*	*10*	**Δ*δ***
δ N1H	6.33	6.36	6.38	6.40	6.42	6.44	6.47	6.49	6.52	6.57	6.62	0.29
δ N2H	8.24	8.21	8.18	8.16	8.13	8.10	8.06	8.02	7.99	7.90	7.82	–0.42

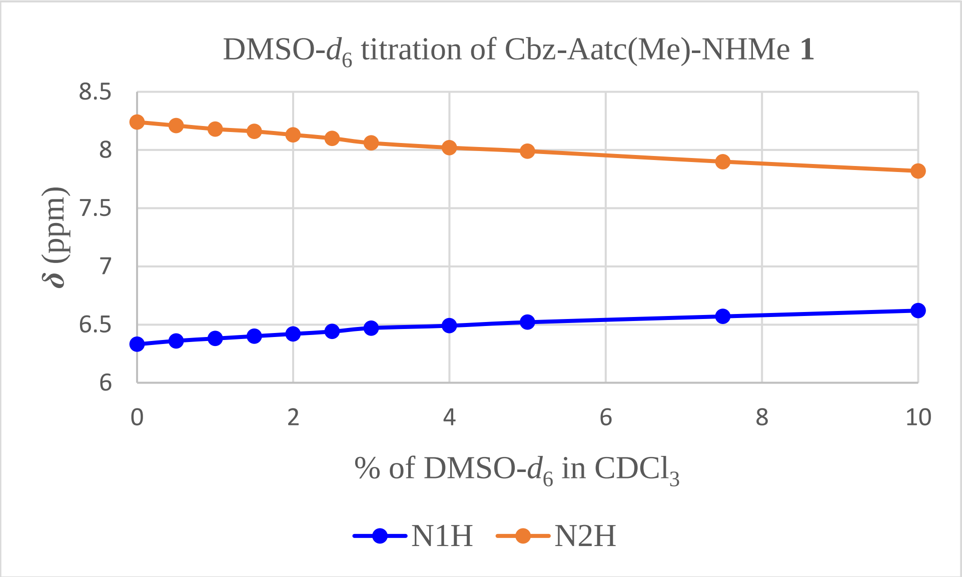

Cbz-[Aatc(Me)]$_2$-NHMe **2**

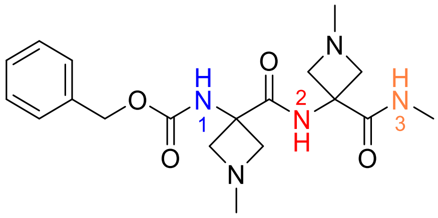

	DMSO-d_6 (%, v/v)											
	0	*0.5*	*1*	*1.5*	*2*	*2.5*	*3*	*4*	*5*	*7.5*	*10*	$\Delta\delta$
δ N1H	6.12	6.16	6.20	6.23	6.27	6.30	6.34	6.40	6.46	6.59	6.70	0.58
δ N2H	9.10	9.06	9.02	8.98	8.94	8.90	8.86	8.79	8.73	8.59	8.48	–0.62
δ N3H	7.96	7.95	7.93	7.91	7.89	7.87	7.86	7.82	7.79	7.71	7.63	–0.33

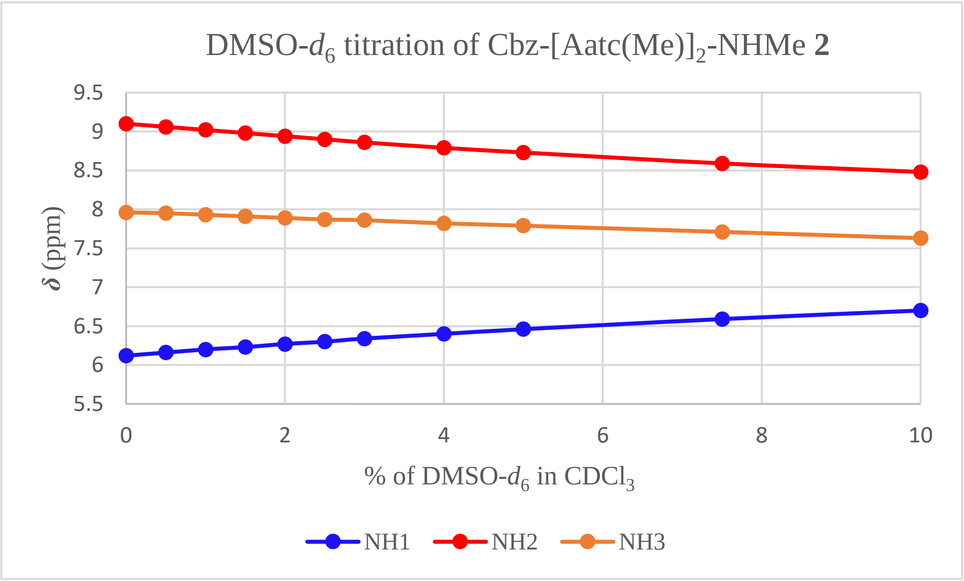

Cbz-[Aatc(Me)]3-NHMe **3**

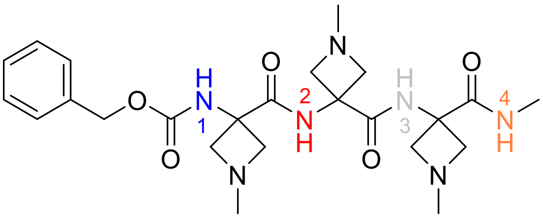

	DMSO-d_6 (%, v/v)											
	0	*0.5*	*1*	*1.5*	*2*	*2.5*	*3*	*4*	*5*	*7.5*	*10*	**Δδ**
δ N1H	6.22	6.32	6.41	6.51	6.59	6.66	6.73	6.84	6.94	7.13	7.17	0.95
δ N2H	8.85	8.80	8.73	8.68	8.63	8.58	8.55	8.49	8.44	8.38	8.34	–0.51
δ N3H	8.48	8.43	8.38	8.33	8.29	8.25	8.21	8.15	8.09	7.98	7.90	–0.58
δ N4H	7.79	7.75	7.70	7.65	7.60	7.56	7.52	7.45	7.40	7.27	7.19	–0.60

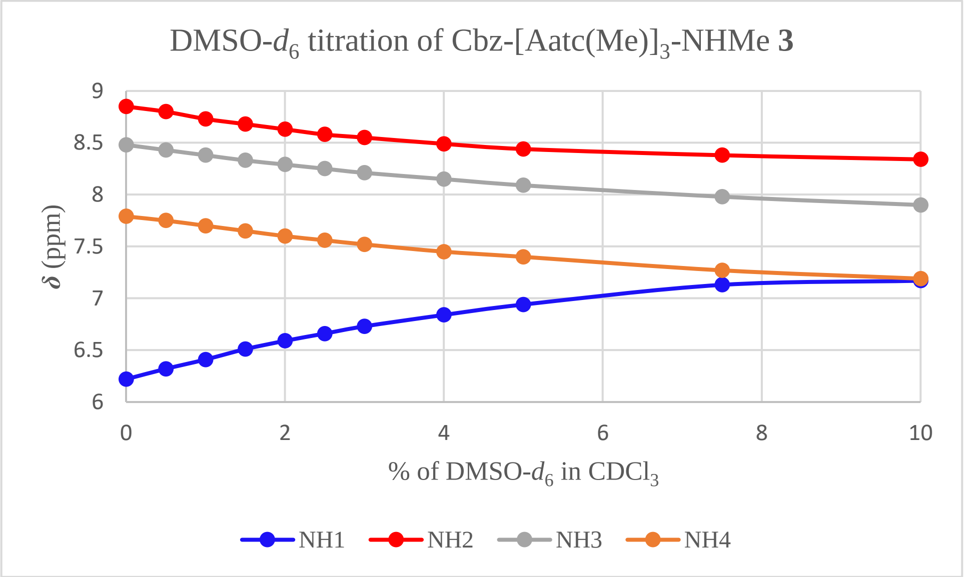

Supplement: Supplementary file 1 [file molecules-28-05048-s001.zip › molecules-2451366-supplementary.pdf]
